# Imaging plant metabolism *in situ*

**DOI:** 10.1093/jxb/erad423

**Published:** 2023-10-27

**Authors:** Patrick J Horn, Kent D Chapman

**Affiliations:** BioDiscovery Institute and Department of Biological Sciences, University of North Texas, Denton TX 76203, USA; BioDiscovery Institute and Department of Biological Sciences, University of North Texas, Denton TX 76203, USA; French Alternative and Atomic Energy Commission, France

**Keywords:** Biochemistry, chemical imaging, desorption ionization mass spectrometry (DESI), mass spectrometry imaging (MSI), matrix-assisted laser desorption/ionization (MALDI), metabolism, metabolome, primary metabolism, spatial maps, specialized metabolism

## Abstract

Mass spectrometry imaging (MSI) has emerged as an invaluable analytical technique for investigating the spatial distribution of molecules within biological systems. In the realm of plant science, MSI is increasingly employed to explore metabolic processes across a wide array of plant tissues, including those in leaves, fruits, stems, roots, and seeds, spanning various plant systems such as model species, staple and energy crops, and medicinal plants. By generating spatial maps of metabolites, MSI has elucidated the distribution patterns of diverse metabolites and phytochemicals, encompassing lipids, carbohydrates, amino acids, organic acids, phenolics, terpenes, alkaloids, vitamins, pigments, and others, thereby providing insights into their metabolic pathways and functional roles. In this review, we present recent MSI studies that demonstrate the advances made in visualizing the plant spatial metabolome. Moreover, we emphasize the technical progress that enhances the identification and interpretation of spatial metabolite maps. Within a mere decade since the inception of plant MSI studies, this robust technology is poised to continue as a vital tool for tackling complex challenges in plant metabolism.

## Teaser

The objective of this review is to provide a timely update on how mass spectrometry imaging is used to visualize plant metabolism *in situ* .

## Introduction Mass spectrometry imaging: a brief overview

The evaluation of a plant metabolome usually begins with the extraction of the mixtures of molecules from some tissue(s) of a plant in an appropriate solvent compatible with further separation and/or analysis by mass spectrometry (MS) (Mushtaq *et al.*, 2014). The extraction process is often optimized for the target class or category of metabolites to be studied, and invariably mixes the metabolites uniformly in a solvent extract as a starting point for metabolomics analysis. Although this approach facilitates the comprehensive qualitative and quantitative analysis of the metabolome (Alseekh *et al.*, 2021; Aharoni *et al.*, 2023), it also completely disrupts the spatial location of those metabolites in the original tissue sample. In contrast, mass spectrometry imaging (MSI) provides an approach for the analysis of metabolite compositions *in situ*, directly in tissue sections or on plant parts (Boughton *et al.*, 2016; Sturtevant *et al.*, 2016). This general MSI approach has been adapted for a variety of applications in plant metabolism studies, using various ionization sources coupled to high-resolution mass spectrometers, as detailed in other recent reviews (Hu *et al.*, 2021; Unsihuay *et al.*, 2021; Dong and Aharoni, 2022; Yin *et al.*, 2022; Borisjuk *et al.*, 2023).

**Fig. 1.**
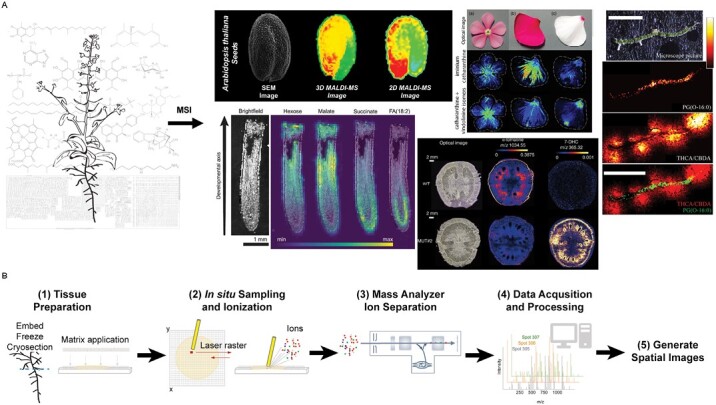
Overview of MSI workflow. There are several steps to generating high- (A) quality spatial metabolism maps highlighted here using the (B) workflow for matrix-assisted laser desorption/ionization mass spectrometry imaging (MALDI-MSI). Tissues to be imaged are prepared by embedding in a suitable medium (e.g. gelatin), freezing, and cryosectioning cross- and/or longitudinal sections (e.g. 30–50 μm). Tissue sections are adhered to a glass slide followed by application of a chemical matrix uniformly applied using a spray directed at the tissue surface. A laser of defined spot size (typically 5–50 μm) is directed at each tissue spot and rastered in discrete steps to cover the entire section to be imaged. Laser impact on the matrix-tissue surface releases ions that are then directed into the mass analyzer for ion separation and detection (e.g. Orbitrap, time-of-flight, Fourier-transform ion cyclotron resonance or FT-ICR). Chemical data containing mass spectra at each tissue spot are then processed using specialized software to identify compounds, quantify their abundance, and generate spatial images. Five representative MSI studies are highlighted to illustrate spatial heterogeneity of metabolites: (top left) lipids in *Arabidopsis thaliana* seeds (graphical abstract, reprinted from Sturtevant *et al.*, 2017 with permission from Elsevier), (bottom left) sugars, organic acids, and lipids in maize roots (modified from T. Zhang *et al.*, 2023), (middle top) alkaloids in periwinkle petals [reproduced from Dutkiewicz *et al.* (2021), with permission], (middle bottom) vitamins in engineered tomato fruit (modified from Li *et al.*, 2022), (right) lipid marker and Δ9-THCA in cannabis sugar leaves [reproduced from Lorensen *et al.* (2023b), with permission]. Additional details are described within the text and in corresponding articles. The metabolic map image is from the Plant Metabolic Network website (Hawkins *et al.*, 2021). All structures generated using ChemDraw V20.

One of the most widely employed MSI techniques involves matrix-assisted laser desorption/ionization (MALDI), and can be accomplished under vacuum or at atmospheric pressure depending upon the specific capabilities of the ionization source (Liu *et al.*, 2022; Zhu *et al.*, 2022). Several high-quality reviews have detailed critical specimen preparation and analysis (Dong *et al.*, 2016; Bokhart *et al.*, 2017; Buchberger *et al.*, 2018; Sturtevant *et al.*, 2021; Caleb Bagley *et al.*, 2023), with a general workflow for MALDI-MSI shown in Fig. 1. Briefly, MALDI-MSI can be performed on essentially any plant tissue sections. Most often samples are flash-frozen in an embedding medium (e.g. gelatin), sectioned on a cryo-microtome (e.g. 10–50 μm in thickness), and then lyophilized to preserve the subcellular organization of all small molecules. A compatible chemical matrix [e.g. 2,5-dihydroxybenzoic acid (DHB)] is then applied to the surface of the tissue section enabling metabolite ionization, with high-resolution mass spectra collected at each location on a tissue section. The final tissue distributions of all selected metabolites can be reconstructed in two dimensions with commercially available or open-source software. The spatial resolution is dependent upon laser spot and raster step size, with 1–10 μm spatial resolution achievable with lasers on most contemporary commercial instruments (Hansen and Lee, 2018). However, most studies in plants routinely use spatial resolutions of 20–50 μm (i.e. around cellular resolution) that balance sampling time and metabolite signal. Chemical mass-to-charge (*m/z*) resolution varies among high-resolution mass spectrometers, but resolutions of >1 000 000 for lipid samples are possible with Fourier-transformed ion cyclotron resonance (FTICR) MS (Tiquet *et al.*, 2022), making it feasible for experimental plans to involve isotope labeling of hundreds of isotopomer species.

While MALDI is the most used ionization method, desorption electrospray ionization (DESI)-MSI has been increasingly used in plant imaging studies. DESI does not use a chemical matrix but instead relies on a charged solvent for ionization at ambient conditions, enabling the analysis of additional plant metabolites (Ferreira *et al.*, 2019; Xue *et al.*, 2019; He *et al.*, 2022). Although DESI can be applied directly to a plant’s surface, thereby minimizing sample pre-treatment, most plant DESI studies still rely on sample embedding, freezing, and cryo-sectioning. Imaging plant metabolites can be challenging due to tissue fragility and high water content, as well as tissues with thick epidermal layers that cannot easily be penetrated by soft ionization techniques. Tissue imprinting offers one approach for addressing these issues while maintaining spatial integrity. While the absence of a chemical matrix reduces errors in metabolite spatial assignment, DESI typically operates at much lower spatial resolutions (50–250 μm) and lower ion sensitivity relative to MALDI. As will be illustrated below, while most recent plant MSI studies have used MALDI instead of DESI, both techniques have contributed to unique insights in plant metabolite imaging.

## Expanding the spatial metabolome coverage

In the past decade, an increasing number of metabolites have been spatially mapped *in situ* (Table 1 with additional details in Supplementary Table S1). Early applications of MSI targeted readily ionizable molecules in primary metabolism, such as glycerolipids, amino acids, and carbohydrates, with fewer reports on specialized metabolism [also known as secondary metabolites or natural products metabolites, as reviewed elsewhere (Lee *et al.*, 2012; Boughton *et al.*, 2016; Sturtevant *et al.*, 2016; Qin *et al.*, 2018)]. However, recent reports have suggested that the technology is increasingly used to study specialized metabolism, thereby drastically expanding the number of metabolites profiled. Although the entire plant kingdom’s metabolome is thought to lie in the range 0.2–1 million molecules, a given plant species may still produce several thousand distinct compounds (Fang *et al.*, 2019). In this section, we review progress towards chemical imaging of the plant metabolome, highlighting recent studies in the last 5 years as summarized in Table 1. The subsections below are organized by metabolite category where practical. While many of these studies analyzed multiple metabolite classes, not all metabolites mapped will be described. MSI remains limited in the number of simultaneous metabolites analyzed within a single biological sample, requiring additional technical advances (discussed further in the Conclusions). While it is difficult to accurately quantify the number of metabolites covered to date, it is no doubt still much less than conventional metabolomics studies performed on tissue extracts. Overall, however, the chemical diversity of metabolites is highly encouraging in terms of expanding metabolite coverage and providing technical conditions for future studies. Finally, it also appears that MSI instrumentation is increasingly accessible to diverse research groups, supporting its utility as a core technique and providing continual insights into *in situ* metabolism.

**Table 1.** Representative plant metabolites analyzed by mass spectrometry imaging

Metabolite class	Metabolites^*a*^	Plant tissue	Reference	Method^*b*^
Lipids	Triacylglycerols (TAGs), phosphatidylcholines (PCs)	Pennycress seed (*Thlaspi arvense* L)	Johnston *et al.* (2022); Romsdahl *et al.* (2021)	MALDI
	TAGs, PCs	Rapeseed (*Brassica napus*)	Lu *et al.* (2018)	MALDI
	PC- and TAG-cyclopropane fatty acids (FAs)	Camelina seed (*Camelina sativa)*	Yu *et al.* (2018)	MALDI
	TAGs, wax esters	Jojoba seed (*Simmondsia chinensis*)	Sturtevant *et al.* (2020)	MALDI
	TAGs with hydroxy FAs	Castor seed (*Ricinus communis* L.)	Sturtevant *et al.* (2019)	MALDI
	PCs	Barrel medic nodule (*Medicao truncatula*)	Dokwal *et al.* (2021)	MALDI
	PCs, lysophospholipids, phosphatidylglycerol (PG)	Tomato leaf (*Solanum lycopersicum*)	Veličković *et al.* (2021)	MALDI
	PCs, lysoPLs, phosphatidylethanolamine (PE), phosphatidic acid (PA), sulfoquinovosyl diacylglycerol (SQDG)	Barley root (*Hordeum vulgare* L.)	Sarabia *et al.* (2018)	MALDI
	PA, PE, PG, phosphatidylinositol (PI)	Arabidopsis leaf (*Arabidopsis thaliana*)	Mugume *et al.* (2022)	MALDI
	Oxylipins, FAs, ergosterol, glycerolipids	Wheat grain (*Triticum* spp.)	Righetti *et al.* (2022)	AP-MALDI
	Stigmastanol	Carrot root (*Daucus carota*)	Xiang *et al.* (2022)	AP-MALDI
Carbohydrates	Sucrose and non-sucrose disaccharides	Onion bulb (*Allium cepa*)	Zhan *et al.* (2021)	MALDI
	Hexoses, sorbitol, sucrose	Apple fruit (*Malus domestica*)	Horikawa *et al.* (2019)	MALDI
	Hexoses, sucrose	Strawberry fruit (*Fragaria × ananassa*)	Wang *et al.* (2021); Enomoto, (2021)	MALDI DESI
	(Enzymatically degraded) cellulose, hemicellulose	Maize stem (*Zea mays*)	Arnaud *et al.* (2020)	MALDI
	Sucrose	Grapevine leaf (*Vitis vinifera*)	Maia *et al.* (2022)	MALDI
Amino acids	Cys, Asn, GABA, Gln, Lys	Strawberry fruit (*Fragaria × ananassa*)	Nizioł *et al.* (2019)	SALDI
	Ala, Asn, Gly, Gln Leu/Ile, Val	Maize root (*Zea mays)*	O’Neill and Lee (2020)	MALDI
	Leu, Asn, Pro, His, Arg, Trp	Lentil seedling (*Lens culinaris* L)	Y.-X. Zhang *et al.* (2023)	AP-MALDI
	Spermidine, spermine	Soybean seed (*Glycine max*)	Sagara *et al.* (2020)	AP-MALDI
	Spermidine, spermine	Danshen root (*Salvia miltiorrhiza*)	Sun *et al.* (2022)	MALDI
	*N*1,*N*10-diferuloylspermidine	Pineapple fruit (*Ananas comosus*)	Suarez *et al.* (2023)	MALDI
	Aconitate, (iso)citrate, succinate, fumarate, malate	Maize root (*Zea mays*)	T. Zhang *et al.* (2023)	DESI
	Malate, citrate	Arabidopsis seedling (*Arabidopsis thaliana*), liverwort seedling (*Marchantia polymorpha*)	Gomez‐Zepeda *et al.* (2021)	MALDI
	Pyruvate, lactate, 2-ketobutyrate, maleate/fumarate, oxaloacetate, malate	Strawberry fruit (*Fragaria × ananassa*)	Enomoto (2021)	DESI
	Malate, citrate, gluconate	Notoginseng root (*Panax notoginseng*)	Sun *et al.* (2021)	MALDI
	Malate, maleate, citrate	Banlangen root (*Isatis tinctoria* L.)	Nie *et al.* (2021)	AP-MALDI
	Malate, citrate	Mango fruit (*Mangifera indica* L.)	Zhao *et al.* (2022)	AFAI
Phenolics	Liquiritigenin, apigenin, naringenin, luteolin, dihydrokaempferol, daidzein, quercetin, taxifolin, kaempferol, isorhamnetin, myricetin, catechin, quercetin 3-β-d-glucoside, baicalin, rutin	Litchi seed (*Litchi chinensis* Sonn.)	Y. Liu *et al.* (2023)	MALDI
	Quercetin, kaempferol, isorhamnetin	Rapeseed stem (*Brassica napus*)	Krysa *et al.* (2023)	MALDI
	Caffeic acid, rosmarinic acid, other phenolic acids	Danshen stem, root, leaf, flower (*Salvia miltiorrhiza*)	Tong *et al.* (2022)	DESI
	Flavonoid aglycones, biflavonoids, flavonoid glycosides, biginkgosides	Ginkgo leaf (*Ginkgo biloba*)	Li *et al.* (2018)	MALDI
	Pinoresinol, phillygenin, forsythoside A, forsythoside E, rutin, caffeic acid	Weeping Forsythia dried fruit (*Forsythia suspensa*)	Jing *et al.* (2022)	MALDI
	Puerarin and derivatives, daidzin, mirificin, ambocin, hesperidin, ononin	*Puerariae* sp. dried root (*Puerariae lobata* and *P. thomsonii*)	Guo *et al.* (2023)	AFA-DESI
	Nobiletin, tangeretin, tetramethoxyflavone, and feruloylputrescine, guaiacol	Citrus leaf (*Citrus sinensis-limonia*)	de Moraes Pontes *et al.* (2020)	DESI
	Apigenin, cannaflavin A, cannaflavin B, cannaflavin C, kaempferol, luteolin, orientin, quercetin, vitexin, isovitexsin	Cannabis leaf (*Cannabis sativa*)	Lorensen *et al.* (2023b)	AP-MALDI
Alkaloids	Strictosidine, reserpine and derivatives, ajmalicine and derivatives, ajmaline and derivatives, serpentine	Devil pepper root, stem, leaf, fruit (*Rauvolfia tetraphylla* L.)	Mohana Kumara *et al.* (2019)	DESI
	Reserpine and rescinnamine, and associated biosynthetic intermediates	Devil pepper root, stem, leaf, fruit (*Rauvolfia tetraphylla* L.)	Lorensen *et al.* (2023a)	AP-MALDI
	Atharanthine, vindolinine, serpentine, vindoline, anhydrovinblastine	Periwinkle petal (*Catharanthus roseus*)	Dutkiewicz *et al.* (2021)	SALDI
	Gelsemine-, koumine-, gelsedine, humantenine-, yohimbine, sapargine-type alkaloids	Heartbreak grass stem, root, leaf (*Gelsemiume legans*)	Z.-H. Wu *et al.* (2022)	DESI
	Arecoline, arecaidine, caffeine, cotinine, guvacine, guvacoline, hordenine, sophoridine, trigonelline, vicine	Areca fruit (Areca catechu)	J. Wu *et al.* (2022)	MALDI
	(Pseudo)ephedrine, methyl(pseudo)ephedrine	Ephedra shoot (*Ephedra sinica*)	Yun *et al.* (2021)	DART
	Atharanthine, vindolinine, serpentine, vindoline, and anhydrovinblastine	Peyote crown and stem (*Lophophora williamsii*)	Lin *et al.* (2023)	AP-MALDI
	Cocaine, truxilline, benzoylecgonine, cinnamoylcocaine	Coca plant (*Erythroxylum coca*)	dos Santos *et al.* (2021)	MALDI/LDI
	(Dehydro)tomatine and dervatives, (dehydro) esculeoside A	Tomato fruit (*Solanum lycopersicum*)	Dong *et al.* (2020); Kazachkova *et al.* (2021)	MALDI
	Lupanine, 13-hydroxylupanine, angustifoline	Narrow-leafed lupin seed (*Lupinus angustifolius*)	Otterbach *et al.* (2019)	MALDI
	Tomatine and derivatives, lycoperoside A-C and H	Cherry tomato fruit (*Lycopersicon esculentum*)	Bednarz *et al.* (2019)	MALDI
	Solasodine and derivatives, chaconine	Black nightshade fruit (*Solanum nigrum*)	Bednarz *et al.* (2019)	MALDI
	Tomatidenol, soladulcidine and derivatives	Bittersweet nightshade fruit (*Solanum dulcamara*)	Bednarz *et al.* (2019)	MALDI
Terpenes	Vitexilactone, vitetrifolin B/E/F, rotundifuran	Chaste tree fruit (*Vitex agnus-castus* L.)	Heskes *et al.* (2018)	MALDI
	Carnosol and tanshinone plus pathway intermediates, other diterpenes	*S. grandifolia* and Danshen root (*Salvia grandifolia* and *S. miltiorrhiza*)	Y.-X. Zhang *et al.* (2023)	AP-MALDI
	Kaurane diterpenes, xylopic acid	Ethiopian pepper fruit (*Xylopia aethiopica*)	Kyekyeku *et al.* (2020)	MALDI
	Platycodin D, platycodin D3, platycoside E	Balloon flower root (*Platycodon grandiflorum*)	Tang *et al.* (2023)	MALDI
	Carnosol, kahweol, lactaroviolin, squalene, toxoids	Taxus leaf (*Taxus mairei*)	Zhan *et al.* (2023)	MALDI
	Momilactone-A/B, phytocassane-A–E	Thai rice leaf (*Oryza sativa*)	Komkleow *et al.* (2021)	MALDI
	Soyasaponins	Soybean root nodules (*Glycine max*)	Agtuca *et al.* (2020)	LAESI
	Nobilomethylene, dendronobilin F/K, rupestonic acid G, isopetasol, dendroside G, dendroterpene C	Noble dendrobium stem (*Dendrobium nobile*)	Q. Liu *et al.* (2023)	MALDI
Vitamins and pigments	(Vit E) tocopherols, tocotrienols	Upland cotton seed (*Gossypium hirsutum*)	Salimath *et al.* (2021)	MALDI
	Vit A1, Vit B1, Vit B6, Vit C	Raw and dried persimmon fruit (*Diospyros kaki*)	Shikano *et al.* (2020)	MALDI
	7-Dehydrocholesterol (provitamin D3), cholesterol	Tomato fruit (*Solanum lycopersicum*)	Li *et al.* (2022)	MALDI
	Anthocyanins	Strawberry fruit (*Fragaria × ananassa*)	Wang *et al.* (2021)	MALDI
	Betalains, chlorophyll *a*	Tobacco leaf (*Nicotiana benthamiana*)	Dong *et al.* (2020)	MALDI
	β-Carotene	Carrot root (*Daucus carota*)	Xiang *et al.* (2022)	AP-MALDI
	Polyacetylenes	Cangzhu root (*Atractylodes lancea*)	Jiang *et al.* (2022)	DESI/PI
Cannabinoids	Δ9-THCA, CBNA	Cannabis leaf (*Cannabis sativa*)	dos Santos *et al.* (2019)	MALDI/LDI
	Δ9-THCA, CBN(A), CBE(A), CBGA	Cannabis leaf (*Cannabis sativa*)	Lorensen *et al.* (2023b)	AP-MALDI
Glucosinolates	Indol-3-ylmethyl glucosinolate (GSL), 4-methylthiobutyl GSL, methylthiooctyl GSL, and other GSLs	Arabidopsis leaf (*Arabidopsis thaliana*)	Morikawa-Ichinose *et al.* (2020)	MALDI
	Isatindigoside F GSL and other GSLs	Banlangen root (*Isatis tinctoria* L.)	Nie *et al.* (2021)	DESI
Hormones	Brassinosteroid, salicylic acid, 1-aminocyclopropane-1-carboxylic acid, abscisic acid, cytokinin auxin	Rice root (*Oryza sativa*)	Shiono and Taira (2020)	Nano-PALDI
Nucleotide bases	Guanine, adenine, adenosine	Banlangen root (*Isatis tinctoria* L.)	Nie *et al.* (2021, 2022)	DESI AP-MALDI

^*a*^ Not all metabolites are listed for each study. Studies are generally categorized by predominant and/or unique metabolites imaged.

^*b*^ Techniques: matrix-assisted laser desorption/ionization (MALDI), atmospheric pressure-MALDI (AP-MALDI), surface-assisted laser desorption/ionization (SALDI), desorption electrospray ionization (DESI), air flow-assisted ionization (AFAI), direct analysis in real-time (DART), DESI with post-photoionization assembly (DESI/PI).

### Lipids

Many early MSI studies were focused on plant lipids because they are easily ionizable molecules via MALDI (e.g. using a DHB matrix) and abundant throughout many plant tissue types (Horn *et al.*, 2012, 2013; Horn and Chapman, 2014). Lipids have numerous biological functions, such as membrane compartmentation, intracellular communication, plant defense, and physical hydrophobic barriers (Li-Beisson *et al.*, 2013). The heterogenous spatial distributions observed in oilseed tissues were largely unexpected, as recently reviewed (Borisjuk *et al.*, 2023) and briefly highlighted here. MSI has substantially enhanced our understanding of the spatial precursor–product relationships among membrane and storage lipids [i.e. phosphatidylcholine (PC) and triacylglycerol (TAG), respectively]. For example, MSI of pennycress (an emerging bioenergy cover crop) seed embryos labeled with ^13^C (see ‘Spatial maps of metabolism using stable isotope labeling’) revealed complex spatial and temporal, tissue-specific labeling patterns of PC and TAG (Romsdahl *et al.*, 2021). Enhancing oil content and tailoring the fatty acid (FA) composition of oilseeds is a longstanding goal within the lipid research community (Horn and Benning, 2016; Park *et al.*, 2021). In two independent, comparative imaging studies of high- versus low-oil lines in pennycress (Johnston *et al.*, 2022) and rapeseed (Lu *et al.*, 2018), the extensive heterogeneity of major TAG and PC molecular species was clearly evident; however, the spatial profiles of high-oil lines were relatively similar to those of low-oil lines. Additional differences to explain the total oil variations became evident by combining MSI with transcriptomics and conventional metabolomics, which supports the use of integrated approaches.

Some oilseeds accumulate uncommon (or unusual) FAs with important industrial and nutritional properties (Ohlrogge *et al.*, 2018). For example, MSI of castor bean, which can accumulate up to 90% hydroxy-FAs, showed heterogeneous tissue distribution of mono-, di-, and tri-hydroxy-TAGs in the embryo and endosperm (Sturtevant *et al.*, 2019). Likewise, in jojoba seeds, wax esters (rarely stored in high amounts in other seeds) accumulated in the cotyledons whereas TAG accumulated in the embryonic axis (Sturtevant *et al.*, 2020). In both studies, integration of MSI with transcriptomics suggested tissue-specific mechanisms and primary metabolic pathways for storage lipid accumulation of selected classes and species. Imaging uncommon FAs in their host plants can offer valuable insights into metabolic bottlenecks, as it has been challenging to engineer agronomically superior oilseeds to accumulate these FAs. In metabolically engineered oilseeds, novel spatial patterns often emerge that represent metabolic changes in endogenous (and introduced) pathways, such as the production of cyclopropane FAs or very long chain polyunsaturated FAs in camelina seeds (Usher *et al.*, 2017; Yu *et al.*, 2018). Collectively, MSI continues to be valuable in understanding the evolution of seed biochemistry and informing metabolic engineering strategies.

MSI of lipids is not restricted to oilseeds and has expanded to most lipid classes. For example, PC molecular species were revealed to have a heterogenous distribution in nodules of the legume Medicago (Dokwal *et al.*, 2021). The distributions changed in response to phosphate-deficient conditions, revealing a potential role for lipid remodeling in phosphate stress within legume–rhizobia interactions. In tomato leaves subjected to physical wounding, MSI showed variable lipid patterns in response to wounding especially in lysophospholipids (which are not commonly imaged due to potential formation via hydrolysis and technical artifacts). LysoPCs were abundant in the injured zone while some lysophosphatidylglycerol (lysoPG) species accumulated in the apex of the injured zone or were depleted (Veličković *et al.*, 2021). These results led to a proposed mechanism of differential spatial hydrolysis of PC and PG in response to wounding. MSI analysis of wheat grains subjected to a fungal disease demonstrated a rapid accumulation of several defense metabolites, including oxylipins in the endosperm consistent with the activation of the 13-lipoxygenase pathway after pathogen assault (Righetti *et al.*, 2022). Several membrane lipid classes profiled in barley roots under salt stress showed distinct profile shifts in molecular species of PC and lysoPC, phosphatidic acid (PA), phosphatidylethanolamine (PE), and sulfoquinovosyldiacylglycerol (SQDG) (Sarabia *et al.*, 2018). Alternatively, in a study on the role of autophagy in Arabidopsis leaves, most phospholipid molecular species in autophagy mutants and under different nitrogen replete/deplete conditions showed unchanged spatial profiles (Mugume *et al.*, 2022). This was surprising considering changes observed in individual lipids from total leaf extracts. However, spatial imaging revealed findings otherwise lost in extract-based analysis; that is, the lipid responses in cells (within sections imaged) must be triggered simultaneously in response to autophagic status. Many studies continue to highlight the role of lipid remodeling in abiotic and biotic stress responses (Yu *et al.*, 2021), where MSI can play an important role in addressing spatial metabolic alterations and mechanisms.

### Carbohydrates (sugars)

Carbohydrates are primary photosynthates with a multitude of physiological functions in plants, such as energy and transport, structural building blocks, developmental and stress signaling responses, and flavor indicators (Pigman, 2012). While MSI can detect most monosaccharides and disaccharides using DHB or compatible matrices, other non-invasive chemical imaging methods, such as MRI, are particularly powerful for visualizing the *in situ* distribution of sugars (Borisjuk *et al.*, 2023). Sweetness is a major factor in consumer preference for many fruits. MSI of different maturity stages within strawberry sections showed accumulation of hexoses (glucose and fructose) and sucrose during ripening (Wang *et al.*, 2021). While hexoses were preferentially located in the pith and receptacle structures, sucrose was more uniformly detected throughout the sections and more abundant in the red maturity stage. Similar hexose and sucrose spatial distributions of mature strawberries were observed using DESI-MSI, along with unique profiles of many other metabolite classes (Enomoto, 2021). The spatial distribution of specific sugars is probably linked to the regulation of ripening through abscisic acid (ABA; along with other metabolites such as organic acids and anthocyanins). In contrast, for apple, where sorbitol is the primary carbohydrate transported from leaves to fruits, this sugar alcohol accumulated preferentially at the center of the fruit whereas sucrose was higher in the cortex (Horikawa *et al.*, 2019). As excessive sorbitol accumulation leads to an abiotic stress disorder called ‘watercore’ (Itai, 2015), additional MSI studies may be beneficial to examining mutants in sorbitol transport.

Resolving isomers in MSI can be difficult, and many times an *m/z* is associated with sucrose when in fact it may be one of several disaccharide isomers. Tandem MS has been used to resolve sucrose and non-sucrose disaccharide isomers in cross-sections of onion bulb, showing distinct isomer patterns in inner versus outer epidermis, results that may reveal additional functional or metabolic roles of disaccharides (Zhan *et al.*, 2021). Polysaccharides are predominantly used for storage and assembly of cell walls in essentially all plant tissues, but their size and chemical linkages render polysaccharides difficult for ionization and detection by current MSI methods. Mapping the distribution of enzymatically degraded cell wall polysaccharides offers an alternative approach, as shown in maize stems (Arnaud *et al.*, 2020). Imaging cellulose, mixed linked β-glycans, and heteroxylans showed distinct patterns within lignified and non-lignified tissues which may help better address the recalcitrance of producing cellulosic biofuels. Recently, grapevine leaf discs infected with the fungal pathogen *Plasmopara viticola* showed the accumulation of sucrose mainly on the veins of infection sites, contributing to new knowledge on the fungal infection structures (Maia *et al.*, 2022). In this study, MSI facilitated the observations of spatial changes in the carbohydrate distribution that is associated with pathogen interaction, and also provided evidence for the authors to hypothesize that the pathogen itself may have gained access to the metabolite for completing its life cycle. Future studies that focus on the detection of sugars associated with signaling and developmental cues such as trehalose 6-phosphate could expand the applications of MSI in the field of carbohydrates.

### Amino acids

In addition to building blocks for protein synthesis, many amino acids are precursors for specialized metabolites and have active roles in plant development and stress responses (Trovato *et al.*, 2021). Free amino acids typically have poor ionization efficiency for MALDI-MSI analysis; however, on-tissue derivatization with coniferyl aldehyde improves the ionization efficiency (Manier *et al.*, 2014) such that all 20 canonical amino acids have been detected and most visualized *in situ* (O’Neill and Lee, 2020). For example, MSI of young roots within maize B73 and Mo17 inbred lines showed a development-associated shift in amino acid signal from the cortex to the root center, and an eventual decrease in overall tissue signal. These spatial profiles are consistent with the transport of certain amino acids from the root to other parts of the maize plant. Furthermore, spatial differences between the inbred lines may point to differences in contributions of direct metabolic synthesis in roots versus transport. The inbred lines also had differences in abundance and localization of amino acids, with hybrid offspring inheriting spatial profile characteristics from both parents, suggesting that spatial metabolic profiling may include biomarkers for breeding programs.

In germinating seedlings of lentil, spatial differences were observed for multiple amino acids from 12 h to 72 h post-germination, hypothesized to be a reflection of protease-mediated hydrolysis of seed storage proteins for germination and seedling establishment (Y.-Z. Zhang *et al.*, 2023). Profiling of amino acids has also been enhanced using a matrix of cationic silver nanoparticles (Nizioł *et al.*, 2019). In strawberry fruits, differences in spatial distributions of amino acids suggests that there is cell-specific metabolic regulation of several amino acid pathways, for example (i) achene-enriched (containing the seed), nitrogen-rich Gln, Lys, and Asn as an energy source for germination; (ii) periphery-enriched, the non-proteinogenic amino acid γ-aminobutyric acid (γ-GABA) important for tissue protection; and (iii) dispersive, Cys the most abundant biothiol in strawberry for oxidative protection. Future studies would benefit from continuing to improve the detection of amino acids as well as establishing precursor–product pathways with spatially distinct metabolites.

Polyamines are aliphatic polycations derived from amino acids (Arg and ornithine) with numerous roles in plant health and stress responses (Handa *et al.*, 2018). In soybean seed cross-sections, two polyamines (spermidine and spermine) were more abundant in the embryo axis relative to the cotyledons, with the highest concentrations in root and shoot meristem tissues (Sagara *et al.*, 2020). Spatial profiles of metabolites reflect enzymatic activity and regulation, and, here, the spatial profiling of polyamines was consistent with immunohistochemistry and fluorescent microscopy studies of the respective polyamine-producing aminopropyltransferase enzymes (Belda-Palazón *et al.*, 2012). In roots of the medicinal plant *Salvia miltiorrhiza*, spermine and spermidine were enriched in the xylem and outer cortex roots of repeatedly cropped plants relative to other root tissue regions (Sun *et al.*, 2022). The authors were surprised to find that normal cropped roots had much higher polyamine levels, which may explain some of the altered growth physiology observed in continuously cropped plants. Finally, a polyamine derivative *N*1,*N*10-diferuloylspermidine, a potential HMG-CoA reductase (HMGCR) inhibitor with the potential to lower cholesterol biosynthesis, was localized in pineapple tissue near the peel/shell and gradually decreased towards the flesh and ovary (Suarez *et al.*, 2023). Cells on the periphery of plant tissues are exposed to UV light and probably regulate the production of protective metabolites such as polyamines, which may explain the distribution of polyamine derivatives in pineapple fruits. Additional MSI studies of polyamines are likely to yield new insights into plant growth and stress responses.

### Organic acids

Organic acids have important roles in plant physiology as central intermediates in carbon metabolism, pH regulation, and redox regulation (Igamberdiev and Eprintsev, 2016), with many types having been spatially mapped *in situ*. For example, DESI-MSI of maize roots in longitudinal sections showed that tricarboxylic acid (TCA) cycle intermediates were enriched in developmentally distinct regions, with implications for plant growth and development (T. Zhang *et al.*, 2023). While succinate was enriched in the meristem, three TCA intermediates, aconitate, fumarate, and malate, were enriched in the differentiation zone. These results were consistent with tissue-specific gene expression profiles [e.g. genes encoding aconitase (producing aconitate), succinate dehydrogenase (producing fumarate), and fumarase (producing malate) were enriched in the differentiation zone]. Disruption of the normal expression patterns in addition to experiments exogenously treating roots with TCA intermediates showed that metabolite localization was highly predictive of molecular functions in stem cell behavior.

Root extrusion is important for plant nutrition, tolerance to cation toxicity, and plant–microbe interactions (Gomez‐Zepeda *et al.*, 2021). An MSI method developed for visualizing organic acids in roots and root exudates of Arabidopsis seedlings in different environments has enabled new ways of examining how localization of these metabolites is linked to physiological responses. Furthermore, in root cross-sections of the medicinal plants, ginseng and Banlangen, multiple organic acids were enriched in the phloem and medulla rather than the xylem (Nie *et al.*, 2021; Sun *et al.*, 2021). The enrichment patterns of these metabolites in ginseng may be reflective of organic acids contributing to the rhizosphere exudate (Goodwin, 2022). In fruits, organic acids also serve as flavoring agents that impact acidity and organoleptic qualities. In mango cross-sections analyzed by air flow-assisted ionization, it was shown that malate was distributed throughout the slice whereas citrate was enriched in the pulp (Zhao *et al.*, 2022). Given their role in central metabolism, and other pathways, applying isotope labeling MSI methods may yield unique insights into the spatial regulation of these metabolic pathways and storage endpoints.

### Phenolics

Phenolics are a large group of structurally diverse, specialized metabolites ranging from simple phenolic compounds to more complex molecules and polymers. These compounds contribute to diverse functions such as plant–herbivore interactions, antioxidants, and the color, taste, and flavor of many herbs, foods, and plant-derived beverages (Rehab and Amira, 2018). Several studies have used MSI to visualize flavonoid-rich plants with promising pharmacological activity. For example, MALDI-MSI of litchi seed tissue sections using the 2-mercaptobenzothiazole (2-MBT) matrix revealed 15 detectable flavonoids grouped into four tissue-distinct spatial patterns: four embryo-enriched flavonoids (e.g. luteolin), three cotyledon-enriched (e.g. rutin), seven distributed throughout both embryo and cotyledon (e.g. apigenin, catechin, quercetin), and one in the testa and cotyledon periphery (e.g. taxifolin) (Y. Liu *et al.*, 2023). Each of these unique tissue enrichment patterns may be attributed to their proposed specialized functional roles in litchi seed and a result of regulation at pathway branch points of shared phenolic substrates.

Similarly, a DESI-MSI study of diverse tissues including roots, flowers, stems, and leaves from Chinese medicinal herb *S. miltiorrhiza* showed phenolic acids abundant in the root cortex and vasculature, in contrast to the abietane diterpene, tanshinone, restricted to the periderm (Tong *et al.*, 2022). Standard metabolomics is an important tool for pathway elucidation. The integration of MSI data can provide spatial context to complex pathways and, in some cases, help address multiple biosynthetic routes. For example, there are multiple possible metabolic pathways for the biosynthesis of rosmarinic acid. MSI showed similar co-localization of two potential substrates—caffeic acid and danshensu—that, when combined with metabolomics-based ^13^C trace studies, suggested this route as the core biosynthesis pathway to produce rosmarinic acid in *Salvia* species.

Other recent MSI studies on medicinal plants have spatially mapped diverse phenolics in *Cannabis sativa* leaves (discussed below with ‘Other metabolites’) (Lorensen *et al.*, 2023b), *Ginkgo biloba* leaves (Li *et al.*, 2018), dried fruits of *Forsythia suspensa* (Jing *et al.*, 2022), dried roots of two *Radix puerariae* species (Guo *et al.*, 2023), and *Paeonia* sp. roots (Li *et al.*, 2021). Finally, imaging of phenolics is not restricted to medicinal plants and is likely to be important to understanding abiotic and biotic interactions. For example, MSI of rapeseed treated with Nod factor-based biofertilizers (flavonoid-induced signaling molecules produced by rhizobia in legume root nodule formation) for testing their impact on non-legumes showed an alteration in stem flavonoid patterns, with quercetin and kaempferol derivatives increasing in concentration post-treatment (Krysa *et al.*, 2023). MSI of metabolites may also be used to screen for pathogenicity, for example specific flavonoids (and other metabolites) accumulated in citrus in response to yellow dragon disease (de Moraes Pontes *et al.*, 2020). These studies on flavonoid spatial distribution lay the groundwork for spatial pathway elucidation, trait selection in human health applications, and genetic engineering of specific phenolic compounds in alternative crops.

### Terpenes

Terpenes are a large and diverse class of isoprene-derived compounds with roles in plant defense, cell development, membrane permeability, light harvesting, and protection, as well as contributing to plant fragrance, taste, and pigments (Rehab and Amira, 2018). Relative to other specialized metabolites, many terpenes without polar groups are not easily ionized. However, there are several recent reports on imaging diterpenoids. For example, DESI-MSI revealed the spatial maps of 33 metabolites involved in diterpenoid tanshinone and carnosol biosynthetic pathways in *S. miltiorrhiza* and *S. grandifolia* roots (Xia *et al.*, 2023). Most of the diterpenoid molecules were visualized in the periderm (with similar results of tanshinone to Tong *et al.*, 2022), although some compounds showed differential accumulation between the two related species. In roots of the medicinal plant *Platycodon grandiflorum*, three bioactive oleanane-type triterpenoid saponins were observed enriched in the R2 versus R1 xylem layer of cells, a rather unusual spatial distribution, that led to discovery of a glycosyltransferase involved in the biosynthesis (Tang *et al.*, 2023). In shoot tissues, some terpenes accumulate in leaves while others accumulate in glandular trichomes (along with other specialized metabolites). For example, in the plant that produces compounds for the anti-cancer drug taxol, *Taxus* sp., a few bioactive terpenes accumulated in the spongy mesophyll layer (e.g. toxoids, carnosol) while squalene was located in palisade mesophyll cells (Zhan *et al.*, 2023). MSI of the medicinal plant *Vitex agnus-castus* L. showed the diterpenoid vitexilactone, linked to lowering prolactin in treating female reproductive conditions, localized on the periphery of fruits presumed to be glandular trichomes (Heskes *et al.*, 2018). Other recent studies analyzing terpenes include kaurane diterpenes in Ethiopian pepper (Kyekyeku *et al.*, 2020), triterpenoid soyasaponins in root nodules disrupted in symbiosis (Agtuca *et al.*, 2020), accumulation of diterpenoid phytoalexins in Thai rice in response to blast disease (Komkleow *et al.*, 2021), and distribution of sesquiterpenes in mature *Dendrobium nobile* stem (Q. Liu *et al.*, 2023). Increasing the amounts of (or engineering new tissues to) produce bioactive compounds requires a better understanding of the mechanisms used to naturally accumulate these compounds. Improvements in terpene ionization and identification should be prioritized to broaden our knowledge of the spatial organization of metabolism for this largest class of specialized metabolites in plants.

### Alkaloids

Alkaloids are structurally diverse, specialized metabolites with a heterocyclic ring containing a nitrogen atom. Alkaloids have diverse functions in plants as feeding deterrents, antimicrobial defenses, and germination inhibitors (Jacobowitz and Weng, 2020). Like flavonoids, given their important pharmaceutical, bioactive properties, it is not surprising that many MSI studies have targeted the spatial profiles of alkaloids to characterize these metabolic pathways and their regulation. For example, two recent imaging studies using DESI- and/or MALDI-MSI showed heterogeneous spatial patterns of monoterpenoid indole alkaloids (MIAs) within diverse tissues of *Rauvolfia tetraphylla* (Mohana Kumara *et al.*, 2019; Lorensen *et al.*, 2023a). MIAs were enriched in the early stages of mature fruit, with several compounds restricted to the exocarp (e.g. yohimbine), while ajmaline was largely restricted to the mesocarp (Mohana Kumara *et al.*, 2019). In roots and stems, enrichment was observed for MIAs in distinct tissues: epidermis (e.g. deserpidine), cortex (e.g. ajmalicine), pith only (e.g. reserpine), and epidermis and pith (e.g. serpentine). MIAs were less often detected in leaves. To produce chemically diverse compounds in specific tissues that use similar initial building blocks, such as trypamine (via tryptophan decarboxylation), inherently requires coordinated metabolic regulation and utilization of diverse biochemical reactions (Horn, 2021). Another study focused on using MSI to address the biosynthesis of reserpine, an anti-hypertension and anti-microbial agent (and biochemically related rescinnamine), by analyzing not only the endproduct reserpine (and rescinnamine) but also the theoretical intermediates (Lorensen *et al.*, 2023a). High-resolution MALDI-MSI provided unprecedented detail, detecting most of the proposed intermediates of reserpine in similar cell types among diverse tissues, and this was reinforced using a deuterium-labeled trypamine precursor. Finally, a matrix-free imaging method using tissue imprints on a functionalized TiO_2_ nanowire solid substrate was highly effective in imaging a range of alkaloid pathways in the fragile petals of periwinkle (Dutkiewicz *et al.*, 2021)

Several alkaloids are also addictive drugs, including nicotine, caffeine, morphine, and the lesser reported arecoline. Arecoline accumulates in the fruit of *Areca catechu*. MALDI-MSI [using a 3,4-dimethoxycinnamic acid (DMCA) matrix] revealed heterogeneous patterns of arecoline and other bioactive alkaloids (J. Wu *et al.*, 2022). Understanding how localization impacts bioactivity will be important for therapeutic applications and may inform understanding of tissue-specific functions for these metabolites. Other alkaloid-enriched medicinal plants recently studied by MSI include *Gelsemium elegans* showing differential accumulation in root and stem cell types and insights into alkaloid diffusion and transfer among tissues (Z.H. Wu *et al.*, 2022). Also imaged were the hallucinogenic compound mescaline enriched in epidermal cells of peyote crown and stem (Lin *et al.*, 2023), and cocaine in leaves of coca plant (dos Santos *et al.*, 2021).

Multiple MSI studies have focused on characterizing the spatial distribution of toxic alkaloids associated with fruit and seed bitterness (an adaptive trait to ward off herbivores prior to the ripening process being finished). For example, as tomato fruits ripen, the bitter compound α-tomatine (an important plant defense compound) is exported from the vacuole to the cytosol and enzymatically converted into non-bitter and non-toxic esculeosides (Kazachkova *et al.*, 2021). An MSI study using virus-induced gene silencing (VIGS) of the α-tomatine transporter showed a dramatic increase in α-tomatine and a decrease in esculeoside A in virus-infected regions. MSI is powerful in studying parallel pathways. In this manner, using RNAi to silence the conversion of dehydrotomatine to α-tomatine (with dehydrotomatine subsequently converted to dehydroesculeoside during ripening) showed that both metabolites were abundant in fruit epidermis and pericarp, but α-tomatine was higher in the wild type and dehydrotomatine higher in the RNAi lines (Dong *et al.*, 2020). In a broader study of the *Solanaceae* family, structurally diverse alkaloids were profiled by MSI (Bednarz *et al.*, 2019). In *Solanum lycopersicum*, the major alkaloids showed distinct spatial patterns in different plant tissues (e.g. fruits versus seed versus stalk versus flower), while in *S. nigrum* and *S. dulcamar* dramatic shifts were observed in alkaloid distribution during fruit ripening. Advanced tissue-specific developmental profiling, such as single-cell RNA-seq, would probably reveal elaborate gene expression profiles that enable these metabolite distributions. Bitterness is not restricted to the fruit of the *Solanaceae* family. In the legume *Lupinus angustifolius*, toxic quinolizidine alkaloids (QAs) have long been known to accumulate in the seed (Otterbach *et al.*, 2019). MSI was able to visualize these bitter QA compounds in pods (but not seeds) of early developmental stages, whereas upon maturation, the distribution of QAs shifted to seeds (and was absent in pods). These results support a possible mechanism for transport of these bitter QAs, thereby opening up new strategies for engineering new (less bitter) varieties.

Finally, typically MSI studies have focused on increased spatial resolution that can address spatial metabolite patterns at the cellular or even organellar levels (Hansen and Lee, 2018; Dong *et al.*, 2020). However, high-resolution imaging is time consuming via MALDI-MS and, therefore, alternative methods are needed for imaging large areas, such as whole plants. To address this need, ambient ionization MS was coupled to direct analysis in real-time time-of flight-MS (DART-TOF-MS) on the medicinal plant *Ephedra* sp. (Yun *et al.*, 2021). Here, using low resolution imaging, ~1 mm spot size, the alkaloids (pseudo)ephedrine and methyl(pseudo)ephedrine were visualized. In stems, the alkaloids were enriched on the inner section (versus the outer section). The side branches (commonly used for medicine) were enriched in these alkaloids relative to the main stem, with concentration gradients decreasing towards the branch tips. Whole-plant chemical imaging is powerful for studying plant physiology and biochemistry, as revealed using other non-invasive imaging approaches (Borisjuk *et al.*, 2023). These MSI studies within alkaloids signify a promising path for future alkaloid pathway discovery, localization, and engineering.

### Vitamins and pigments

Plant vitamins are essential for metabolism as enzymatic cofactors and their roles in redox chemistry (Asensi-Fabado and Munné-Bosch, 2010). Cottonseed oil is naturally rich in vitamin E components but contains little or no tocotrienols (Salimath *et al.*, 2021). To improve the oil value, tocotrienols were produced through genetic engineering of upland cotton. MSI showed that engineered tocotrienols accumulated throughout the transgenic embryos while α- and γ-tocopherol were enriched in the cotyledon or embryonic axis, respectively, despite similar upstream substrates. This location contrasted with the polyphenol gossypol located in embryo gland cells. Introducing changes in metabolic pathways via engineering requires careful consideration of endogenous spatial distributions and pathway fluxes, as well as promoters for gene expression of introduced enzymes and other levels of metabolic regulation. Using similar genome editing approaches to address human vitamin D deficiencies and the poor vitamin D content in plants, tomato fruits were engineered to produce 7-dehydrocholesterol (7-DHC or provitamin D3) (Li *et al.*, 2022). Imaging 7-DHC (and small amounts of downstream cholesterol) showed a relatively equal distribution in the flesh and peel of tomatoes. Surprisingly, α-tomatine levels were also reduced, with shifts in its metabolic distribution providing insights into these integrated pathways. In another study, the provitamin A β-carotene within carrots was widely found in the phloem and cambium regions, while it was less common in the metaxylem and protoxylem regions, pointing to the roles of carotenoid transport and metabolite accumulation (Xiang *et al.*, 2022). Lastly, many plants are processed prior to human consumption. MSI of raw versus dried persimmon fruit tissues showed a dramatic change in localization and relative amounts of vitamins: lipid-soluble vitamin A1 increased in the pericarp, water-soluble vitamin B1 and B6 increased in the moist mesocarp, and vitamin C underwent photodegradation (Shikano *et al.*, 2020). Considering the prevalence of nutrient deficiencies and poor dietary habits of many humans, using MSI should assist in efforts for breeding, fortification, and processing of vitamin-rich foods.

Plant pigments are important in light harvesting, photoprotection, and overall growth and development of plants, as well as serving as visible signals to attract insects, birds, and animals for pollination and seed dispersal (Fernández-López *et al.*, 2020). MSI is frequently used to visualize pigment distribution in plants. For example, the red anthocyanins of strawberries were mainly located in the periphery of fruit at late maturity stages associated with antioxidant roles (Wang *et al.*, 2021). In the medicinal plant *Atractylodes lancea*, the color of the rhizoma secretory cavities is often associated with quality, and this color varies widely among accessions (Jiang *et al.*, 2022, Preprint). MSI found that three polyacetylenes were probably contributing to the red color and could serve as a biomarker for trait selection. Although the biosynthesis pathways for these polyacetylenes have not been elucidated (the genome for *A. lancea* has not been reported), MSI suggests that a transcriptomics analysis of these secretory cavities may reveal gene candidates. Finally, the natural colorants betalains were heterologously produced in tobacco through transient overexpression of key genes, showing accumulation of pigment at the site of infection in contrast to naturally produced chlorophyll which is widely distributed (Dong *et al.*, 2020). Transient expression studies are powerful for testing and confirming metabolic pathways. The importance of color (and associated functions) necessitates further studies on pigment biosynthesis and localization via MSI.

### Other metabolites

Cannabinoids are of broad pharmaceutical interest, with recent demand increases driving trait optimization of the host plant *Cannabis sativa* and renewed interest in the biosynthesis of these compounds (Lorensen *et al.*, 2023b). An initial MSI study of cannabis leaves determined that 2-mercaptobenzothiazole (MBT) was the optimal matrix for MALDI detection of the acidic cannabinoids Δ9-THCA (Δ-9-tetrahydrocannabinolic acid) and cannabinolic acid (CBNA). Punctate accumulation patterns were observed, albeit at lower resolution. A follow-up study combining the lower resolution DESI-MSI (both tissue imprints and direct analysis) with higher resolution MALDI-MSI [using a 1,5-diaminonaphthalene (DAN) matrix] largely confirmed the accumulation of cannabinoids within glandular trichomes (by comparison with a chloroplast-specific lipid showing the contour of the leaf). This study also expanded the number of cannabinoids of interest detected, such as THCA, CBNA, CBN, CBE(A), and CBGA, to understanding synergistic effects of bioactive compounds. In contrast to the discrete cannabinoid localization, several flavonoids also appeared more dispersed throughout the leaf. It will be interesting to observe how future engineering designs in alternative hosts may address the location of production for cannabinoid biosynthesis.

For most MSI studies, compounds are present and stable in the tissue of interest under multiple sampling conditions. However, for sulfur-containing glucosinolates with roles in plant defense and plant storage, it was observed that these compounds localized to inner regions of leaves and decreased markedly under darkness (Morikawa-Ichinose *et al.*, 2020). Several glusoinolates were also detected in the dried root of the medicinal plant *Isatis tinctoria*, for example isatindigoside F which accumulated mostly in phloem (Nie *et al.*, 2021). Like variations observed in field versus controlled environmental studies, it will be important to prioritize sampling conditions for all MSI studies.

Nucleosides and nucleobases are not commonly imaged due to their lower molecular weight; however, a few studies have captured spatial maps. For example, in dried root of *Isatis tinctoria*, guanine, adenine, and adenosine were located in the cambium and outer areas of the phloem (Nie *et al.*, 2022). In a related study on *Isatis* root, guanine was located almost exclusively in the root xylem (Nie *et al.*, 2021). In addition to roles in nucleic acid biosynthesis, nucleoside triphosphates (dNTPs) are essential molecules for energy coupling, use building blocks shared with other metabolic pathways, and themselves may be precursors for downstream metabolites. Chemical imaging of these compounds may inform differences in spatial utilization among plant tissues.

Plant hormones are notoriously difficult to image via MALDI-MSI due to matrix effects on low-abundant small molecules (<500 *m/z*). However, nanoparticle-assisted LDI (nano-PALDI)-MSI has enabled the mapping of multiple hormones in root cross-sections of rice (Shiono and Taira, 2020). In the elongation zone, brassinosteroid, salicylic acid, and the ethylene precursor ACC (1-aminocyclopropane-1-carboxylic acid) were broadly distributed, whereas ABA and cytokinin (CK) were higher in the outer root cells relative to the stele, and auxin (IAA) was detected in the epidermis, cortex, and stele. MSI is particularly well suited to address questions regarding hormone localization given that the metabolic pathway location for synthesis is often distant from the site of action. Imaging the developmental and response patterns for hormones in plant tissues more broadly will probably support major research advances in the future given the wide range of physiological responses regulated by these diverse molecules.

Finally, plants grown in natural environments are exposed to exogenous metabolites, xenobiotics, and contaminants (Singh *et al.*, 2016). In recent studies, MSI has revealed (i) in maize roots, the fungal mycotoxin (aflatoxin B1) infiltrated the root vascular cells resulting in the disruption of multiple metabolic pathways and their spatial distributions (Righetti *et al.*, 2021); (ii) in carrot roots, prolonged exposure to a phthalate ester organic pollutant showed its accumulation in vascular cells and associated changes in plant metabolites (Xiang *et al.*, 2022); (iii) in apples, an applied fungicide imazalil penetrated from the peel to the inner region during storage (Pereira *et al.*, 2019); (iv) in wheat leaves, penetration and accumulation of the fungicide azoxystrobin in the cells occurred around the vascular bundle; and (v) in cabbage leaves, visualization enabled a proposed translocation mechanism for carrier-mediated modified pesticides (Wu *et al.*, 2020). MSI enables a fresh technical approach for examining the incorporation and spatial distribution of exogenous compounds and their impact on plant metabolism.

## Spatial maps of metabolism using stable isotope labeling

LC/MS-based approaches to analyze stable isotope-labeled tissues has become commonplace to trace metabolites and characterize flux through specific metabolic pathways (Freund and Hegeman, 2017; Allen and Young, 2020). Less commonly employed is the application of MSI to the localization of metabolites following stable isotope labeling. However, recent reports highlight both the promise and challenge of combining MSI with ^13^C labeling approaches, especially becoming more frequent in biomedical applications (Grey *et al.*, 2021). The challenges are both analytical and computational, but advances in MS instrumentation and computational workflows have made it possible to map the locations of complex metabolic pathway metabolites, even where there may be many potential isotopomers of each metabolite in a single ^13^C labeling experiment. For example, in studies aimed at assessing metabolic heterogeneity in glioblastoma tumors, mice fed [^13^C]glucose were subjected to MSI of brain sections (Schwaiger-Haber *et al.*, 2023). Here, a combination of DESI- and MALDI-MSI was used to evaluate metabolic pathways induced in tumors. FA synthesis was elevated 8-fold in tumors over nearby healthy cells, and a specific enhancement of elongation of FAs was noted in glioblastoma tumors. In other work, the metabolism of surfactant in lung tissues was examined by MSI, where application of [^13^C]PC species enabled visualization of lipid remodeling in lung tissues (Ellis *et al.*, 2021). Other stable isotopes, including ^2^H and ^15^N, can be useful in MSI approaches to visualize metabolism. By way of example, MSI (using secondary ion MS, SIMS) demonstrated that the metabolic utilization of [^2^H]glucose and [^15^N]glutamine by different tumors *in situ* was remarkably heterogenous among individual tumors (Zhang *et al.*, 2020). In addition to monitoring metabolism, MSI by SIMS of [^13^C]thymidine and [^15^N]thymidine has been used to image DNA in stem cells (McMahon and Lechene, 2021). Variations of this multi-isotope imaging mass spectrometry (MIMS) using a focused ion beam to generate secondary ions has remarkable lateral resolution of <50 nm and has been used to image processes at subcellular resolution (Gyngard and Steinhauser, 2019), such as protein turnover by lysosomes (Narendra *et al.*, 2019) and lipid sorting to neuronal lipid droplets (Bailey *et al.*, 2015). In plants, nanoSIMS has been used in parallel with NMR and MALDI-MSI to examine drought-tolerant mechanisms in plant roots of *Piper* sp., *Hibiscus rosa sinensis*, and *Clitoria fairchildiana* roots (Honeker *et al.*, 2022). NanoSIMS of [^13^C]pyruvate-labeled roots showed different ^13^C distribution patterns, associated with carbohydrate storage, in the presence versus absence of its rhizosphere. The rhizosphere is an important contributor to drought responses in many plants.

**Fig. 2.**
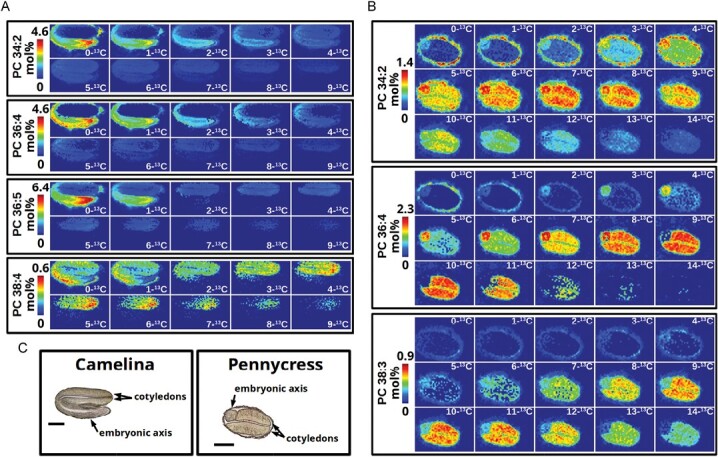
Example of using isotope labeling to enhance MSI in plants. This is a reproduction of fig. 4 and modified legend from Romsdahl *et al.* (2021). Images are shown for ^13^C-MSI of phosphatidylcholine molecular species from a developing camelina embryo (A) and a developing pennycress embryo (B) after isotopically labeling with [U-^13^C]glucose for 64 h and 120 h, respectively. Brightfield images (C) of camelina and pennycress embryo sections indicate anatomy and localization of cotyledon and embryonic axis tissues (scale bar=500 μm). Isotopologs of ^13^C-MSI [e.g. total number of isotopic ^13^C carbons (0–14) incorporated per all acyl chains (34–38 carbons)] are aligned from least to most ^13^C incorporation from left to right and top to bottom. Each set of images for each molecular species is set to a colorimetric scale from dark blue (low abundance) to red (high abundance) representing mol% relative to the total ion count of the imaging data. Molecular species are shown with total number of carbons: total number of double bonds.

Although mostly applied to date to animal tissues, MSI studies of metabolism with stable isotopes are beginning to emerge in plant systems. In one report, siliques of two Brassica species (camelina and pennycress) were fed with [^13^C]glucose, and the isotopologs of PC species (a key intermediate in TAG accumulation) were imaged in developing embryos by MALDI-MSI at 64 h and 120 h after labeling following transport of labeled precursor into seed tissues. These efforts helped shed light on the metabolic processes for the conversion of carbohydrate into storage lipids in plant embryos, particularly in the later stages of glycerolipid assembly. Here differences in FA desaturation and elongation were noted among different tissues in the embryos (Romsdahl *et al.*, 2021). In both plant species, embryos incorporated ^13^C into PC species with different efficiency in cotyledons and embryonic axis tissues of developing seeds (Fig. 2). In addition, in both tissues, ^13^C isotopes of longer chain, more saturated PC species were enriched over those PC species with polyunsaturated species, suggesting that FA elongation proceeds more rapidly in embryos compared with desaturation activities.

It is likely that future studies with stable isotopes will begin to reveal more nuances about the compartmentalization of plant metabolism. Nonetheless, it remains a significant challenge to combine isotope labeling approaches with MALDI-MSI. Matrix interference can obscure spectra of small metabolites, and larger molecules that are more easily resolved by MALDI-MS have increased complexity with more carbon atoms. For example, a typical PC molecular species with one palmitoyl and one linoleoyl acyl chain has 44 carbons, including the head group, so thus up to 44 potential isotopologs that could be labeled with ^13^C. Plants typically have ≥20 molecular species of PC. Further, a difference of 0.009 amu separates the *m/z* of a saturated FA versus an unsaturated FA with two ^13^C isotopes. Hence, while Romsdahl *et al.* (2021) demonstrated the feasibility of MSI and stable isotope labeling in plant embryos, the experimental data from a ^13^C labeling experiment can be extremely complex even when just focusing on one class of a particular phospholipid. Most probably advances in computational capabilities and the incorporation of artificial intelligence applications into MS data analysis will make these challenges more tractable, and we will see more examples of isotope labeling studies of spatial metabolism in plant systems in the future.

## Conclusions: the future of spatial metabolome imaging

Chemical-based, spatial imaging techniques have enabled researchers to see and study plant metabolism as never before (Borisjuk *et al.*, 2023). MSI delivers a powerful spatial snapshot revealing metabolite distribution and relative concentration that has resulted from complex mechanisms of gene expression and metabolism. In the past 5 years, there has been a dramatic expansion in the number of metabolite classes, tissue types, and the plant species that have been examined by MSI (Table 1). Through these studies, common themes continue to emerge that reinforce the utility of MSI approaches for plant metabolism research and suggest the following three key questions/areas for future development.

### Heterogeneous spatial patterns

Why and how are metabolites visualized by MSI consistently organized in heterogeneous spatial distribution patterns *in situ*? For some specialized pathways, the answer is obvious in that plants produce and concentrate molecules where they are needed. However, this phenomenon extends to metabolites involved in primary and specialized metabolism, irrespective of their abundance, and across various tissue types and plant species. Furthermore, MSI has now supplied overwhelming evidence that metabolite heterogeneity is a key contributor in engineering bioproducts in plants, and therefore should be considered to achieve targeted outcomes. Consequently, it is imperative to re-evaluate ways to delineate metabolic pathways. Traditionally, metabolic pathways are depicted as discrete biochemical steps that connect substrates, products, enzymatic reactions, and encoded genes, as exemplified by the Plant Metabolic Network pathways (Hawkins *et al.*, 2021). These representations are invaluable and certainly adequate for many applications. However, they often fail to account for spatial and temporal variations or tissue-specific differences in pathway dynamics. Future investigations, supported by advanced computational techniques and visualization methods, should strive to incorporate these additional dimensions when exploring plant metabolism.

### Pathway discovery and elucidation

Can MSI play a broader, systematic role in metabolic pathway elucidation and discovery? Conventional metabolomics, supported by genetic approaches, is critical for characterizing biochemical pathways. Nevertheless, MSI is increasingly used to examine the spatial profiles of metabolic intermediates and endproducts, providing additional insights into metabolic pathways. In some cases, co-localization of metabolites has suggested contributions of a specific alternative pathway that may have been hidden in analysis of extracts by conventional metabolomics. MSI data are highly insightful when integrated with metabolic maps and tissue-specific transcriptomics. In most cases (but not all), the heterogeneous spatial patterns can be attributed to variations in gene expression patterns for individual biochemical steps or pathways. Furthermore, steady-state isotope labeling of plant tissues visualized by MSI, while complex to analyze, could help link active metabolic pathways and metabolite accumulations *in situ*. Future studies that integrate isotope labeling, single-cell RNA-seq, and single-cell proteomics technologies should enable exciting new findings in the field of plant metabolism.

### Overcoming limitations in identification, quantification, and interpretation

What limitations and technical challenges need to be overcome for MSI to be employed as a routine metabolomics tool? MSI still has limitations as a chemical imaging methodology. Other recent reviews have addressed various limitations in more detail (Dong *et al.*, 2016; Hu *et al.*, 2021; Unsihuay *et al.*, 2021; Dong and Aharoni, 2022; Borisjuk *et al.*, 2023), and a few key limitations are highlighted here. Foremost, like most analytical techniques, sample preparation is key and there is no single methodology that can image all metabolites simultaneously at this time (i.e. the entire plant metabolome). However, an increasing number of proof-of-concept studies for diverse metabolites provide empirical evidence for researchers wishing to incorporate MSI into their analytical toolbox (Table 1). In terms of methods, MALDI-MSI has been far more commonly used than DESI-MSI, contributing to a wider scope of types of metabolites imaged (Supplementary Table S1). Based on these studies, the most common reasons attributed to using MALDI-MSI (versus DESI-MSI) included superior spatial resolution, enhanced metabolite signal, and ionization source availability. DESI-MSI was used more often when imaging fragile tissues and to illustrate technical developments. Across all MALDI studies, >15 types of matrix and nanoparticles were used, with DHB the most common, covering most metabolite classes. Systematic testing and optimization of matrices remains limited in terms of recommendation for use on specific metabolites. Nonetheless, even under ideal sample conditions, MSI is limited in compound identification and verification due to the limited number of ions available for analysis and resolution of isobaric compounds. Advances in MSI sensitivity and resolution [e.g. transmission-mode geometry (t-MALDI-MSI) and laser-induced post-ionization (MALDI-2), (Niehaus *et al.*, 2019)], supported by advanced computational algorithms, should continue to improve the utility of MSI in these areas. While absolute quantification of compounds is difficult, advances have been made incorporating internal standards within chemical matrices (for MALDI) and methods for normalizing differences in ionization efficiencies. In most cases, relative quantification of metabolites remains informative. Finally, MSI raw data and generated images are often complex to interpret and integrate with other multi-omics datasets. Machine learning and artificial intelligence-based algorithms are increasingly used in MSI analysis, and represent a key development that will enhance the processing and interpretation of MSI information (Verbeeck *et al.*, 2020; Boiko *et al.*, 2022; Aharoni *et al.*, 2023; Jetybayeva *et al.*, 2023).

## Supplementary data

The following supplementary data are available at *JXB* online.

Table S1. Representative plant metabolites analyzed by mass spectrometry imaging (extended Table 1).

erad423_suppl_Supplementary_Tables_S1
