# Supplementary material for: Imaging plant metabolism in situ
Source: J Exp Bot. 2023 Oct 27;75(6):1654–70. doi: 10.1093/jxb/erad423 (PMC10938046; doi:10.1093/jxb/erad423)
Supplement: erad423_suppl_Supplementary_Tables_S1 [file erad423_suppl_supplementary_tables_s1.pdf]

Table S1. Representative Plant Metabolites Analyzed by Mass Spectrometry Imaging (Extended Table 1)

Metabolite Class	Metabolites[1]	Plant Tissue	Reference	Method[2]	Spatial Resolution (µm)	MSI Instrument	MALDI Matrix(ces)[3]
Lipids	triacylglycerols (TAGs), phosphatidylcholines (PCs)	pennycress seed (*Thlaspi arvense* L)	(Johnston *et al.*, 2022)<br>(Romsdahl *et al.*, 2021)	MALDI	40	Thermo Scientific MALDI-LTQ-Orbitrap-XL	DHB
	TAGs, PCs	rapeseed (*Brassica napus*)	(Lu *et al.*, 2018)	MALDI	40	Thermo Scientific MALDI-LTQ-Orbitrap-XL	DHB
	PC- and TAG- cyclopropane fatty acids (FAs)	camelina seed (*Camelina sativa)*	(Yu *et al.*, 2018)	MALDI	40	Thermo Scientific MALDI-LTQ-Orbitrap-XL	DHB
	TAGs, wax esters	jojoba seed (*Simmondsia chinensis*)	(Sturtevant *et al.*, 2020)	MALDI	40	Thermo Scientific MALDI-LTQ-Orbitrap-XL	DHB
	TAGs with hydroxy FAs	castor seed (*Ricinus communis* L.)	(Sturtevant *et al.*, 2019)	MALDI	40	Thermo Scientific MALDI-LTQ-Orbitrap-XL	DHB
	PCs	barrel medic nodule (*Medicao truncatula*)	(Dokwal *et al.*, 2021)	MALDI	40	Thermo Scientific MALDI-LTQ-Orbitrap-XL	DHB
	PCs, lysophospholipids, phosphatidylglycerol (PG)	tomato leaf (*Solanum lycopersicum*)	(Veličković *et al.*, 2021)	MALDI	20/75	Bruker Solarix 15T-FTICR and Bruker timsTOF flex	DHB, norharmane
	PCs, lysoPLs, phosphatidylethanolamine (PE), phosphatidic acid (PA), sulfoquinovosyl diacylglycerol (SQDG)	barley root (*Hordeum vulgare* L.)	(Sarabia *et al.*, 2018)	MALDI	30	Bruker SolariX 7T XR hybrid ESI–MALDI–FT–ICR–MS	DHB
	PA, PE, PG, phosphatidylinositol (PI)	Arabidopsis leaf (*Arabidopsis thaliana*)	(Mugume *et al.*, 2022)	MALDI	100	Thermo Scientific MALDI-LTQ-Orbitrap-Discovery	DHB, DAN
	oxylipins, FAs, ergosterol, glycerolipids	wheat grain (*Triticum spp.*)	(Righetti *et al.*, 2022)	AP-MALDI	20	TransMIT AP-SMALDI5 AF-Thermo Fisher Scientific Q Exactive HF	DHB
	stigmastanol	carrot root (*Daucus carota*)	(Xiang *et al.*, 2022)	AP-MALDI	20	Shimadzu MALDI–TOF-MS iMScope TRIO	DHB
Carbohydrates	sucrose and non-sucrose disaccharides	onion bulb (*Allium cepa*)	(Zhan *et al.*, 2021)	MALDI	200	Bruker MALDI-TOF/TOF MS UltrafleXtreme	NEDC
	hexoses, sorbitol, sucrose	apple fruit (*Malus domestica*)	(Horikawa *et al.*, 2019)	MALDI	500	Bruker MALDI-TOF/TOF MS UltrafleXtreme	DHB, CHCA
	hexoses, sucrose	strawberry fruit (*Fragaria x ananassa*)	(Wang *et al.*, 2021)<br>(Enomoto, 2021)	MALDI<br>DESI	200<br>200	Bruker MALDI-TOF/TOF MS UltrafleXtreme<br>Waters 2D DESI-Synapt XS Q-TOF-MS	DHB<br>n/a
	(enzymatically degraded) cellulose, hemicellulose	maize stem (*Zea mays)*	(Arnaud *et al.*, 2020)	MALDI	40	Bruker rapifleX MALDI Tissuetyper MALDI-TOF-MS	DMA+DHB
	sucrose	grapevine leaf (*Vitis vinifera*)	(Maia *et al.*, 2022)	MALDI	200	Bruker SolariX XR 9.4T hybrid ESI-MALDI-FT-ICR-MS	DHB, CHCA, 9-AA
Amino Acids	Cys, Asn, GABA, Gln, Lys	strawberry fruit (*Fragaria x ananassa*)	(Nizioł *et al.*, 2019)	SALDI	100	Bruker Autoflex Speed TOF-MS	^{109}Ag nanoparticles
	Ala, Asn, Gly, Gln Leu/Ile, Val	Maize root (*Zea mays)*	(O'Neill and Lee, 2020)	MALDI	20	Thermo Scientific MALDI-LTQ-Orbitrap-Discovery	CA+gold
	Leu, Asn, Pro, His, Arg, Trp	lentil seedling (*Lens culinaris* L)	(Zhang *et al.*, 2023b)	AP-MALDI	15/45	Shimadzu MALDI–TOF-MS iMScope TRIO	CHCA
	spermidine, spermine	soybean seed (*Glycine max*)	(Sagara *et al.*, 2020)	AP-MALDI	35	TransMIT AP-SMALDI10 AF-Thermo Fisher Scientific Q Exactive HF	DHB
	spermidine, spermine	Danshen root (*Salvia miltiorrhiza*)	(Sun *et al.*, 2022)	MALDI	75	Bruker rapifleX MALDI Tissuetyper MALDI-TOF-MS	DAN, BNDM

	N1, N10-diferuloylspermidine	pineapple fruit (*Ananas comosus*)	(Suarez *et al.*, 2023)	MALDI	200	Bruker Autoflex Speed TOF-MS	CHCA, DHB
Organic Acids	aconitate, (iso)citrate, succinate, fumarate, malate	maize root (*Zea mays*)	(Zhang *et al.*, 2023a)	DESI	80/130	Customized DESI probe with Thermo Scientific MALDI-LTQ-Orbitrap-XL	n/a
	malate, citrate	Arabidopsis seedling (*Arabidopsis thaliana*)<br>liverwort seedling (*Marchantia polymorpha*)	(Gomez-Zepeda *et al.*, 2021)	MALDI	50/200	Waters Synapt G1 HDMS	CHCA
	pyruvate, lactate, 2-ketobutyrate, maleate/fumarate, oxaloacetate, malate	strawberry fruit (*Fragaria x ananassa*)	(Enomoto, 2021)	DESI	200	Waters 2D DESI-Synapt XS Q-TOF-MS	n/a
	malate, citrate, gluconate	notoginseng root (*Panax notoginseng*)	(Sun *et al.*, 2021)	MALDI	100	Bruker rapifleX MALDI Tissuetyper MALDI-TOF-MS	DHB, CHCA, 9-AA
	malate, maleate, citrate	Banlangen root (*Isatis tinctoria* L.)	(Nie *et al.*, 2021)	AP-MALDI	80	Shimadzu MALDI–TOF-MS iMScope	DHAP, DAN
	malate, citrate	mango fruit (*Mangifera indica* L.)	(Zhao *et al.*, 2022)	AFAI	100	AFAI-Thermo Fisher Scientific Q Exactive Plus	n/a
Phenolics	liquiritigenin, apigenin, naringenin, luteolin, dihydrokaempferol, daidzein, quercetin, taxifolin, kaempferol, isorhamnetin, myricetin, catechin, quercetin 3-β-d-glucoside, baicalin, rutin	Litchi seed (*Litchi chinensis* Sonn.)	(Liu *et al.*, 2023b)	MALDI	250	Bruker Autoflex Speed TOF-MS	MBT
	quercetin, kaempferol, isorhamnetin	rapeseed stem (*Brassica napus*)	(Krysa *et al.*, 2023)	MALDI	55	Waters Synapt G2-*Si* HDMS	CHCA
	caffeic acid, rosmarinic acid, other phenolic acids	Danshen stem, root, leaf, flower (*Salvia miltiorrhiza*)	(Tong *et al.*, 2022)	DESI	100	Waters Xevo G2-XS QTOF	n/a
	flavonoid aglycones, biflavonoids, flavonoid glycosides, biginkgosides	ginkgo leaf (*Ginkgo biloba*)	(Li *et al.*, 2018)	MALDI	50	Bruker SolariX 7T XR hybrid ESI–MALDI–FT–ICR–MS	DHB, CHCA, 9-AA
	pinoresinol, phillygenin, forsythoside A, forsythoside E, rutin, caffeic acid	weeping Forsythia dried fruit (*Forsythia suspensa*)	(Jing *et al.*, 2022)	MALDI	100	Bruker rapifleX MALDI Tissuetyper MALDI-TOF-MS	DAN
	puerarin and derivatives, daidzin, mirificin, ambocin, hesperidin, ononin	*Puerariae* sp. dried root (*Puerariae lobata* and *P. thomsonii*)	(Guo *et al.*, 2023)	AFA-DESI	200	Thermo Fisher Scientific Q Exactive	n/a
	nobiletin, tangeretin, tetramethoxyflavone, and feruloylputrescine, guaiacol	citrus leaf (*Citrus sinensis-limonia*)	(de Moraes Pontes *et al.*, 2020)	DESI	200	Thermo Fisher Scientific Q Exactive	n/a
	apigenin, cannaflavin A, cannaflavin B, cannaflavin C, kaempferol, luteolin, orientin, quercetin, vitexin isovitexsin	cannabis leaf (*Cannabis sativa*)	(Lorensen *et al.*, 2023b)	AP-MALDI	150/200	TransMIT AP-SMALDI5 AF-Thermo Fisher Scientific Q Exactive HF	DAN
Alkaloids	strictosidine, reserpine and derivatives, ajmalicine and derivatives, ajmaline and derivatives, serpentine	devil pepper root, stem, leaf, fruit (*Rauvolfia tetraphylla* L.)	(Mohana Kumara *et al.*, 2019)	DESI	250	Prosolia Omni Spray-Thermo Scientific MALDI-LTQ-Orbitrap-XL	n/a
	reserpine and rescinnamine, and associated biosynthetic intermediates	devil pepper root, stem, leaf, fruit (*Rauvolfia tetraphylla* L.)	(Lorensen *et al.*, 2023a)	AP-MALDI	15/20	TransMIT AP-SMALDI5 AF-Thermo Fisher Scientific Q Exactive HF	DHB
	atharanthine, vindolinine, serpentine, vindoline, anhydrovinblastine	periwinkle petal (*Catharanthus roseus*)	(Dutkiewicz *et al.*, 2021)	SALDI	250	Bruker Autoflex Speed TOF-MS	TiO_2 nanowires
	gelsemine- , koumine-, gelsedine, humantenine-, yohimbine, sapargine-type alkaloids	heartbreak grass stem, root, leaf (*Gelsemiume legans*)	(Wu *et al.*, 2022b)	DESI	50	Waters Xevo G2-XS QTOF	n/a
	arecoline, arecaidine, caffeine, cotinine, guvacine, guvacoline, hordenine, sophoridine, trigonelline, vicine	Areca fruit (Areca catechu)	(Wu *et al.*, 2022a)	MALDI	100	Bruker Autoflex Speed TOF-MS	DMCA

	(pseudo)ephedrine, methyl(pseudo)ephedrine	Ephedra shoot (*Ephedra sinica*)	(Yun *et al.*, 2021)	DART	1000	IonSense DART- JEOL Accu-TOF-MS	n/a
	atharanthine, vindolinine, serpentine, vindoline and anhydrovinblastine	peyote crown and stem (Lophophora williamsii)	(Lin *et al.*, 2023)	AP-MALDI	30	Shimadzu MALDI–TOF-MS iMScope QT	DTCB
	cocaine, truxilline, benzoylecgonine, cinnamoylcocaine	coca plant (*Erythroxylum coca*)	(dos Santos *et al.*, 2021)	MALDI/LDI	150	Bruker SolariX XR 9.4T hybrid ESI-LDI/MALDI-FT-ICR-MS	CHCA, DHB, MBT
	(dehydro)tomatine and dervatives, (dehydro) esculeoside A	tomato fruit (*Solanum lycopersicum*)	(Dong *et al.*, 2020; Kazachkova *et al.*, 2021)	MALDI	60/150	Bruker SolariX 7T XR hybrid ESI–MALDI–FT–ICR–MS	DHB
	lupanine, 13-hydroxylupanine, angustifoline	narrow-leafed lupin seed (*Lupinus angustifolius*)	(Otterbach *et al.*, 2019)	MALDI	30	TransMIT AP-SMALDI10 AF-Thermo Fisher Scientific Q Exactive HF	DHB
	tomatine and derivatives, lycoperoside A-C and H	cherry tomato fruit (*Lycopersicon esculentum*)	(Bednarz *et al.*, 2019)	MALDI	50	Bruker MALDI-TOF/TOF MS UltrafleXtreme	DHB
	solasodine and derivatives, chaconine	black nightshade fruit (*Solanum nigrum*)	(Bednarz *et al.*, 2019)	MALDI	50	Bruker MALDI-TOF/TOF MS UltrafleXtreme	DHB
	tomatidenol, soladulcidine and derivatives	bittersweet nightshade fruit (*Solanum dulcamara*)	(Bednarz *et al.*, 2019)	MALDI	50	Bruker MALDI-TOF/TOF MS UltrafleXtreme	DHB
Terpenes	vitexilactone, vitetrifolin B/E/F, rotundifuran	chaste tree fruit (*Vitex agnus-castus* L.)	(Heskes *et al.*, 2018)	MALDI	40	Bruker SolariX 7T XR hybrid ESI–MALDI–FT–ICR–MS	DHB
	carnosol and tanshinone plus pathway intermediates, other diterpenes	*S. grandifolia* and Danshen root (*Salvia grandifolia* and *S. miltiorrhiza*)	(Zhang *et al.*, 2023b)	AP-MALDI	15/45	Shimadzu MALDI–TOF-MS iMScope TRIO	CHCA
	kaurane diterpenes, xylopic acid	Ethiopian pepper fruit (*Xylopia aethiopica*)	(Kyekyeku *et al.*, 2020)	MALDI	15	Thermo Scientific MALDI-LTQ-Orbitrap-XL	CHCA
	platycodin D, platycodin D3 platycoside E	balloon flower root (*Platycodon grandiflorum*)	(Tang *et al.*, 2023)	MALDI	50	Bruker timsTOF fleX	DHB
	carnosol, kahweol, lactaroviolin, squalene, toxoids	Taxus leaf (*Taxus mairei*)	(Zhan *et al.*, 2023)	MALDI	20	Bruker timsTOF flex MALDI-2	DHB+DHAP
	momilactone-A/B, phytocassane-A-E	Thai rice leaf *(Oryza sativa)*	(Komkleow *et al.*, 2021)	MALDI	50	MALDI-SpiralTOF (JMS-S3000)	Fe_3O_4
	soyasaponins	soybean root nodules (*Glycine max*)	(Agtuca *et al.*, 2020)	LAESI	200	Custom LAESI-Waters Synapt G2S	n/a
	nobilomethylene, dendronobilin F/K, rupestonic acid G, isopetasol, dendroside G, dendroterpene C	noble dendrobium stem (*Dendrobium nobile*)	(Liu *et al.*, 2023a)	MALDI	20	Bruker AutoFlex Speed MALDI TOF	MBT
Vitamins and Pigments	(Vit E) tocopherols, tocotrienols	upland cotton seed (*Gossypium hirsutum*)	(Salimath *et al.*, 2021)	MALDI	80	Thermo Scientific MALDI-LTQ-Orbitrap-XL	DHB
	Vit A1, Vit B1, Vit B6, Vit C	raw and dried persimmon fruit (*Diospyros kaki*)	(Shikano *et al.*, 2020)	MALDI	120	Bruker rapifleX MALDI Tissuetyper MALDI-TOF-MS	CHCA
	7-dehydrocholesterol (provitamin D3), cholesterol	tomato fruit (*Solanum lycopersicum*)	(Li *et al.*, 2022)	MALDI	105	Waters Synapt G2-*Si* HDMS	DHB
	anthocyanins	strawberry fruit (*Fragaria x ananassa*)	(Wang *et al.*, 2021)	MALDI	200	Bruker MALDI-TOF/TOF MS UltrafleXtreme	DHB
	betalains, chlorophyll a	tobacco leaf (*Nicotiana benthamiana*)	(Dong *et al.*, 2020)	MALDI	60	Bruker SolariX 7T XR hybrid ESI–MALDI–FT–ICR–MS	DHB
	β-carotene	carrot root (*Daucus carota*)	(Xiang *et al.*, 2022)	AP-MALDI	20	Shimadzu MALDI–TOF-MS iMScope TRIO	DHB
	polyacetylenes	Cangzhu root (*Atractylodes lancea*)	(Jiang *et al.*, 2022)	DESI/PI	200	Agilent 6224 Accurate-Mass TOF	n/a
Cannabinoids	Δ9-THCA, CBNA	cannabis leaf (*Cannabis sativa*)	(dos Santos *et al.*, 2019)	MALDI/LDI	150	Bruker SolariX XR 9.4T hybrid ESI-LDI/MALDI-FT-ICR-MS	CHCA, DHB, MBT
	Δ9-THCA, CBN(A), CBE(A), CBGA	cannabis leaf (*Cannabis sativa*)	(Lorensen *et al.*, 2023b)	AP-MALDI	150/200	TransMIT AP-SMALDI5 AF-Thermo Fisher Scientific Q Exactive HF	DAN

Glucosinolates	indol-3-ylmethyl glucosinolate (GSL), 4-methylthiobutyl GSL, methylthiooctyl GSL, and other GSLs	arabidopsis leaf (*Arabidopsis thaliana*)	(Morikawa-Ichinose *et al.*, 2020)	MALDI	50	Shimadzu AXIMA Confidence MALDI-TOF	9-AA
	isatindigoside F GSL and other GSLs	Banlangen root (*Isatis tinctoria* L.)	(Nie *et al.*, 2021)	DESI	80	Waters 2D DESI-Synapt XS Q-TOF-MS	n/a
Hormones	brassinosteroid, salicylic acid, 1-aminocyclopropane-1-carboxylic acid, abscisic acid, cytokinin auxin	rice root (*Oryza sativa*)	(Shiono and Taira, 2020)	Nano-PALDI	15	Bruker MALDI-TOF/TOF MS UltrafleXtreme	Fe-nanoparticles
Nucleotide bases	guanine, adenine, adenosine	Banlangen root (*Isatis tinctoria* L.)	(Nie *et al.*, 2021; Nie *et al.*, 2022)	DESI<br>AP-MALDI	80<br>80	Waters 2D DESI-Synapt XS Q-TOF-MS<br>Shimadzu MALDI–TOF-MS iMScope	n/a<br>DHAP, DAN

[1]Not all metabolites are listed for each study. Studies are generally categorized by predominant and/or unique metabolites imaged.

[2]Techniques: matrix-assisted laser desorption/ionization (MALDI), atmospheric pressure-MALDI (AP-MALDI), surface-assisted laser desorption/ionization (SALDI), desorption electrospray ionization (DESI), air flow-assisted ionization (AFAI), direct analysis in real-time (DART), DESI with post-photoionization assembly (DESI/PI)

[3]Matrices: 2,5-dihydroxybenzoic acid (DHB), β-Carboline (norharmane), 1,5-diaminonaphthalene (DAN), N-(1-naphthyl) ethylenediamine dihydrochloride (NEDC), alpha-cyano-4-hydroxycinnamic acid (CHCA), N,N-dimethylaniline (DMA), 9-aminoacridine (9-AA), 4-hydroxy-3-cinnamaldehyde (CA), 1,1′-binaphthyl-2,2′-diamine (BNDM), 2′, 5′-dihydroxyacetophenone (DHAP), trans-2-[3-(4-tert-butylphenyl)-2-methyl-2-propenylidene]malononitrile (DTCB), 2-mercaptobenzothiazole (MBT), 3,4-dimethoxycinnamic acid (DMCA)

Reference

Agtuca BJ, Stopka SA, Evans S, Samarah L, Liu Y, Xu D, Stacey MG, Koppenaal DW, Paša-Tolić L, Anderton CR. 2020. Metabolomic profiling of wild-type and mutant soybean root nodules using laser-ablation electrospray ionization mass spectrometry reveals altered metabolism. The Plant Journal **103**, 1937-1958.

Arnaud B, Durand S, Fanuel M, Guillon F, Méchin V, Rogniaux H. 2020. Imaging Study by Mass Spectrometry of the Spatial Variation of Cellulose and Hemicellulose Structures in Corn Stalks. Journal of Agricultural and Food Chemistry **68**, 4042-4050.

Bednarz H, Roloff N, Niehaus K. 2019. Mass Spectrometry Imaging of the Spatial and Temporal Localization of Alkaloids in Nightshades. Journal of Agricultural and Food Chemistry **67**, 13470-13477.

de Moraes Pontes JG, Vendramini PH, Fernandes LS, de Souza FH, Pilau EJ, Eberlin MN, Magnani RF, Wulff NA, Fill TP. 2020. Mass spectrometry imaging as a potential technique for diagnostic of Huanglongbing disease using fast and simple sample preparation. Scientific reports **10**, 13457.

Dokwal D, Romsdahl TB, Kunz DA, Alonso AP, Dickstein R. 2021. Phosphorus deprivation affects composition and spatial distribution of membrane lipids in legume nodules. Plant Physiology **185**, 1847-1859.

Dong Y, Sonawane P, Cohen H, Polturak G, Feldberg L, Avivi SH, Rogachev I, Aharoni A. 2020. High mass resolution, spatial metabolite mapping enhances the current plant gene and pathway discovery toolbox. New Phytologist **228**, 1986-2002.

dos Santos NA, de Almeida CM, Gonçalves FF, Ortiz RS, Kuster RM, Saquetto D, Romão W. 2021. Analysis of *Erythroxylum coca* Leaves by Imaging Mass Spectrometry (MALDI–FT–ICR IMS). Journal of the American Society for Mass Spectrometry **32**, 946-955.

dos Santos NA, de Souza LM, Pinto FE, de J. Macrino C, de Almeida CM, Merlo BB, Filgueiras PR, Ortiz RS, Mohana-Borges R, Romão W. 2019. LDI and MALDI-FT-ICR imaging MS in *Cannabis* leaves: optimization and study of spatial distribution of cannabinoids. Analytical Methods **11**, 1757-1764.

Dutkiewicz EP, Su C-H, Lee H-J, Hsu C-C, Yang Y-L. 2021. Visualizing vinca alkaloids in the petal of *Catharanthus roseus* using functionalized titanium oxide nanowire substrate for surface-assisted laser desorption/ionization imaging mass spectrometry. The Plant Journal **105**, 1123-1133.

Enomoto H. 2021. Adhesive film applications help to prepare strawberry fruit sections for desorption electrospray ionization-mass spectrometry imaging. Bioscience, Biotechnology, and Biochemistry **85**, 1341-1347.

Gomez-Zepeda D, Frausto M, Nájera-González HR, Herrera-Estrella L, Ordaz-Ortiz JJ. 2021. Mass spectrometry-based quantification and spatial localization of small organic acid exudates in plant roots under phosphorus deficiency and aluminum toxicity. The Plant Journal **106**, 1791-1806.

Guo N, Fang Z, Zang Q, Yang Y, Nan T, Zhao Y, Huang L. 2023. Spatially resolved metabolomics combined with bioactivity analyses to evaluate the pharmacological properties of two *Radix Puerariae* species. Journal of Ethnopharmacology **313**, 116546.

Heskes AM, Sundram TCM, Boughton BA, Jensen NB, Hansen NL, Crocoll C, Cozzi F, Rasmussen S, Hamberger B, Hamberger B, Staerk D, Møller BL, Pateraki I. 2018. Biosynthesis of bioactive diterpenoids in the medicinal plant *Vitex agnus*-castus. The Plant Journal **93**, 943-958.

Horikawa K, Hirama T, Shimura H, Jitsuyama Y, Suzuki T. 2019. Visualization of soluble carbohydrate distribution in apple fruit flesh utilizing MALDI–TOF MS imaging. Plant Science **278**, 107-112.

Jiang D, Liu Z-H, Qi K, Wang H, Zhang C, Zhu L, Zhang Y, Pan Y, Guo L. 2022. Spatial metabolomics reveals potential biomarkers for red secretory cavities in *Atractylodes lancea* natural accessions. Authorea Preprints. doi: 10.22541/au.165752847.75087336/v1. [Preprint].

Jing F, Wang L, Yang M, Wu C, Li J, Shi L, Feng S, Li F. 2022. Visualizing the spatial distribution of functional metabolites in *Forsythia suspensa* at different harvest stages by MALDI mass spectrometry imaging. Fitoterapia **162**, 105285.

Johnston C, García Navarrete LT, Ortiz E, Romsdahl TB, Guzha A, Chapman KD, Grotewold E, Alonso AP. 2022. Effective Mechanisms for Improving Seed Oil Production in Pennycress (*Thlaspi arvense* L.) Highlighted by Integration of Comparative Metabolomics and Transcriptomics. Frontiers in Plant Science **13**.

Kazachkova Y, Zemach I, Panda S, Bocobza S, Vainer A, Rogachev I, Dong Y, Ben-Dor S, Veres D, Kanstrup C, Lambertz SK, Crocoll C, Hu Y, Shani E, Michaeli S, Nour-Eldin HH, Zamir D, Aharoni A. 2021. The GORKY glycoalkaloid transporter is indispensable for preventing tomato bitterness. Nature Plants **7**, 468-480.

Komkleow S, Niyomploy P, Sangvanich P. 2021. Maldi-mass Spectrometry Imaging for Phytoalexins Detection in RD6 Thai Rice. Applied Biochemistry and Microbiology **57**, 533-541.

Krysa M, Susniak K, Kubas A, Kidaj D, Sroka-Bartnicka A. 2023.) MALDI MSI and Raman Spectroscopy Application in the Analysis of the Structural Components and Flavonoids in *Brassica napus* Stem. *Metabolites* 10.3390/metabo13060687.

Kyekyeku JO, Asare-Nkansah S, Bekoe SO, Sezgin S, Adosraku RK, Spiteller M. 2020. MALDI–HRMS imaging and HPLC–HRESI–MSn characterisation of kaurane diterpenes in the fruits of *Xylopia aethiopica* (Dunal) A. Rich (Annonaceae). Phytochemical Analysis **31**, 349-354.
Li B, Neumann EK, Ge J, Gao W, Yang H, Li P, Sweedler JV. 2018. Interrogation of spatial metabolome of *Ginkgo biloba* with high-resolution matrix-assisted laser desorption/ionization and laser desorption/ionization mass spectrometry imaging. Plant, Cell & Environment **41**, 2693-2703.
Li J, Scarano A, Gonzalez NM, D'Orso F, Yue Y, Nemeth K, Saalbach G, Hill L, de Oliveira Martins C, Moran R, Santino A, Martin C. 2022. Biofortified tomatoes provide a new route to vitamin D sufficiency. Nature Plants **8**, 611-616.
Lin J, Yun K, Sun Q, Xiang P, Wu L, Yang S, Dun J, Fu S, Chen H. 2023. How to sample a seizure plant: the role of the visualization spatial distribution analysis of *Lophophora williamsii* as an example. Forensic Sciences Research, owad014.
Liu Q, Huang Y, Linghu C, Xiao J, Gu R. 2023a. Metabolic profiling, in-situ spatial distribution, and biosynthetic pathway of functional metabolites in Dendrobium nobile stem revealed by combining UPLC-QTOF-MS with MALDI-TOF-MSI. Frontiers in Plant Science **13**.
Liu Y, Nie X, Wang J, Zhao Z, Wang Z, Ju F. 2023b. Visualizing the distribution of flavonoids in litchi (*Litchi chinenis*) seeds through matrix-assisted laser desorption/ionization mass spectrometry imaging. Frontiers in Plant Science **14**.
Lorensen MDBB, Bjarnholt N, St-Pierre B, Heinicke S, Courdavault V, O'Connor S, Janfelt C. 2023a. Spatial localization of monoterpenoid indole alkaloids in *Rauvolfia tetraphylla* by high resolution mass spectrometry imaging. Phytochemistry **209**, 113620.
Lorensen MDBB, Hayat SY, Wellner N, Bjarnholt N, Janfelt C. 2023b. Leaves of *Cannabis sativa* and their trichomes studied by DESI and MALDI mass spectrometry imaging for their contents of cannabinoids and flavonoids. Phytochemical Analysis **34**, 269-279.
Lu S, Sturtevant D, Aziz M, Jin C, Li Q, Chapman KD, Guo L. 2018. Spatial analysis of lipid metabolites and expressed genes reveals tissue-specific heterogeneity of lipid metabolism in high- and low-oil *Brassica napus* L. seeds. The Plant Journal **94**, 915-932.
Maia M, McCann A, Malherbe C, Far J, Cunha J, Eiras-Dias J, Cordeiro C, Eppe G, Quinton L, Figueiredo A, De Pauw E, Sousa Silva M. 2022. Grapevine leaf MALDI-MS imaging reveals the localisation of a putatively identified sucrose metabolite associated to *Plasmopara viticola* development. Frontiers in Plant Science **13**.
Mohana Kumara P, Uma Shaanker R, Pradeep T. 2019. UPLC and ESI-MS analysis of metabolites of *Rauvolfia tetraphylla* L. and their spatial localization using desorption electrospray ionization (DESI) mass spectrometric imaging. Phytochemistry **159**, 20-29.
Morikawa-Ichinose T, Miura D, Zhang L, Kim S-J, Maruyama-Nakashita A. 2020. Involvement of BGLU30 in Glucosinolate Catabolism in the *Arabidopsis* Leaf under Dark Conditions. Plant and Cell Physiology **61**, 1095-1106.
Mugume Y, Ding G, Dueñas ME, Liu M, Lee Y-J, Nikolau BJ, Bassham DC. 2022. Complex Changes in Membrane Lipids Associated with the Modification of Autophagy in *Arabidopsis*. *Metabolites* 10.3390/metabo12020190.
Nie L-X, Dong J, Huang L-Y, Qian X-Y, Lian C-J, Kang S, Dai Z, Ma S-C. 2021. Microscopic Mass Spectrometry Imaging Reveals the Distribution of Phytochemicals in the Dried Root of *Isatis tinctoria*. Frontiers in Pharmacology **12**.
Nie L-X, Huang L-Y, Wang X-P, Lv L-F, Yang X-X, Jia X-F, Kang S, Yao L-W, Dai Z, Ma S-C. 2022. Desorption Electrospray Ionization Mass Spectrometry Imaging Illustrates the Quality Characters of *Isatidis Radix*. Frontiers in Plant Science **13**.
Nizioł J, Misiorek M, Ruman T. 2019. Mass spectrometry imaging of low molecular weight metabolites in strawberry fruit (*Fragaria* x *ananassa* Duch.) cv. Primoris with 109Ag nanoparticle enhanced target. Phytochemistry **159**, 11-19.
O'Neill KC, Lee YJ. 2020. Visualizing genotypic and developmental differences of free amino acids in maize roots with mass spectrometry imaging. Frontiers in Plant Science **11**, 639.
Otterbach SL, Yang T, Kato L, Janfelt C, Geu-Flores F. 2019. Quinolizidine alkaloids are transported to seeds of bitter narrow-leafed lupin. Journal of Experimental Botany **70**, 5799-5808.
Righetti L, Gottwald S, Tortorella S, Spengler B, Bhandari DR. 2022. Mass Spectrometry Imaging Disclosed Spatial Distribution of Defense-Related Metabolites in *Triticum* spp. Metabolites **12**, 48.
Romsdahl TB, Kambhampati S, Koley S, Yadav UP, Alonso AP, Allen DK, Chapman KD. 2021.) Analyzing Mass Spectrometry Imaging Data of 13C-Labeled Phospholipids in Camelina sativa and *Thlaspi arvense* (Pennycress) Embryos. *Metabolites* 10.3390/metabo11030148.
Sagara T, Bhandari DR, Spengler B, Vollmann J. 2020. Spermidine and other functional phytochemicals in soybean seeds: Spatial distribution as visualized by mass spectrometry imaging. Food Science & Nutrition **8**, 675-682.
Salimath SS, Romsdahl TB, Konda AR, Zhang W, Cahoon EB, Dowd MK, Wedegaertner TC, Hake KD, Chapman KD. 2021. Production of tocotrienols in seeds of cotton (*Gossypium hirsutum* L.) enhances oxidative stability and offers nutraceutical potential. Plant Biotechnology Journal **19**, 1268-1282.
Sarabia LD, Boughton BA, Rupasinghe T, van de Meene AML, Callahan DL, Hill CB, Roessner U. 2018. High-mass-resolution MALDI mass spectrometry imaging reveals detailed spatial distribution of metabolites and lipids in roots of barley seedlings in response to salinity stress. Metabolomics **14**, 63.
Shikano H, Miyama Y, Ikeda R, Takeshi H, Suda J, Yoshinaga K, Taira S. 2020. Localization analysis of multiple vitamins in dried persimmon (*Diospyros kaki*) using matrix-assisted laser desorption/ionization mass spectrometry imaging. Journal of Oleo Science **69**, 959-964.
Shiono K, Taira S. 2020. Imaging of Multiple Plant Hormones in Roots of Rice (*Oryza sativa*) Using Nanoparticle-Assisted Laser Desorption/Ionization Mass Spectrometry. Journal of Agricultural and Food Chemistry **68**, 6770-6775.
Sturtevant D, Lu S, Zhou Z-W, Shen Y, Wang S, Song J-M, Zhong J, Burks DJ, Yang Z-Q, Yang Q-Y, Cannon AE, Herrfurth C, Feussner I, Borisjuk L, Munz E, Verbeck GF, Wang X, Azad RK, Singleton B, Dyer JM, Chen L-L, Chapman KD, Guo L. 2020. The genome of jojoba (*Simmondsia chinensis*): A taxonomically isolated species that directs wax ester accumulation in its seeds. Science Advances **6**, eaay3240.
Sturtevant D, Romsdahl TB, Yu X-H, Burks DJ, Azad RK, Shanklin J, Chapman KD. 2019. Tissue-specific differences in metabolites and transcripts contribute to the heterogeneity of ricinoleic acid accumulation in *Ricinus communis* L. (castor) seeds. Metabolomics **15**, 6.
Suarez AFL, Juanillo AAB, Sison DCD, Orajay JI, Javier MBB, Baladjay DMS, Yang Y-L, Saludes JP, Dalisay DS. 2023. In situ spatiotemporal mapping of 3-hydroxy-3-methylglutaryl-CoA reductase (HMGCR) inhibitor in pineapple (*Ananas comosus*) fruit tissue by MALDI mass spectrometry imaging. Frontiers in Natural Products **2**.
Sun C, Cui L, Zhou B, Wang X, Guo L, Liu W. 2022. Visualizing the spatial distribution and alteration of metabolites in continuously cropped *Salvia miltiorrhiza* Bge using MALDI-MSI. Journal of Pharmaceutical Analysis **12**, 719-724.
Sun C, Ma S, Li L, Wang D, Liu W, Liu F, Guo L, Wang X. 2021. Visualizing the distributions and spatiotemporal changes of metabolites in *Panax notoginseng* by MALDI mass spectrometry imaging. Journal of Ginseng Research **45**, 726-733.
Tang W, Shi J-J, Liu W, Lu X, Li B. 2023. MALDI Imaging Assisted Discovery of a Di-O-glycosyltransferase from *Platycodon grandiflorum* Root. Angewandte Chemie International Edition **62**, e202301309.
Tong Q, Zhang C, Tu Y, Chen J, Li Q, Zeng Z, Wang F, Sun L, Huang D, Li M. 2022. Biosynthesis-based spatial metabolome of *Salvia miltiorrhiza* Bunge by combining metabolomics approaches with mass spectrometry-imaging. Talanta **238**, 123045.
Veličković D, Chu RK, Henkel C, Nyhuis A, Tao N, Kyle JE, Adkins JN, Anderton CR, Paurus V, Bloodsworth K, Bramer LM, Cornett DS, Curtis WR, Burnum-Johnson KE. 2021. Preserved and variable spatial-chemical changes of lipids across tomato leaves in response to central vein wounding reveals potential origin of linolenic acid in signal transduction cascade. Plant-Environment Interactions **2**, 28-35.
Wang J, Yang E, Chaurand P, Raghavan V. 2021. Visualizing the distribution of strawberry plant metabolites at different maturity stages by MALDI-TOF imaging mass spectrometry. Food Chemistry **345**, 128838.
Wu J, Cui C, Zhao H, Zhou G, Qin L, Li X, Chen L, Wang X, Wan Y. 2022a. In-situ detection and imaging of *Areca catechu* fruit alkaloids by MALDI-MSI. Industrial Crops and Products **188**, 115533.
Wu Z-H, Wang R-Z, Sun Z-L, Su Y, Xiao L-T. 2022b. A mass spectrometry imaging approach on spatiotemporal distribution of multiple alkaloids in *Gelsemium elegans*. Frontiers in Plant Science **13**.
Xiang L, Wang F, Bian Y, Harindintwali JD, Wang Z, Wang Y, Dong J, Chen H, Schaeffer A, Jiang X, Cai Z. 2022. Visualizing the Distribution of Phthalate Esters and Plant Metabolites in Carrot by Matrix-Assisted Laser Desorption/Ionization Imaging Mass Spectrometry. Journal of Agricultural and Food Chemistry **70**, 15311-15320.
Yu X-H, Cahoon RE, Horn PJ, Shi H, Prakash RR, Cai Y, Hearney M, Chapman KD, Cahoon EB, Schwender J, Shanklin J. 2018. Identification of bottlenecks in the accumulation of cyclic fatty acids in camelina seed oil. Plant Biotechnology Journal **16**, 926-938.

Yun N, Kim HJ, Park SC, Park G, Kim MK, Choi YH, Jang YP. 2021.) Localization of Major Ephedra Alkaloids in Whole Aerial Parts of Ephedrae Herba Using Direct Analysis in Real Time-Time of Flight-Mass Spectrometry. *Molecules* 10.3390/molecules26030580.
Zhan L, Huang X, Xue J, Liu H, Xiong C, Wang J, Nie Z. 2021. MALDI-TOF/TOF tandem mass spectrometry imaging reveals non-uniform distribution of disaccharide isomers in plant tissues. Food Chemistry **338**, 127984.
Zhan X, Qiu T, Zhang H, Kailin H, Liang X, Chen C, Wang Z, Wu Q, Wang X, Li X-l. 2023. Mass spectrometry imaging and single-cell transcriptional profiling reveal the tissue-specific regulation of bioactive ingredient biosynthesis in Taxus leaves. Plant Communications **338**, 127984.
Zhang T, Noll SE, Peng JT, Klair A, Tripka A, Stutzman N, Cheng C, Zare RN, Dickinson AJ. 2023a. Chemical imaging reveals diverse functions of tricarboxylic acid metabolites in root growth and development. Nature communications **14**, 2567.
Zhang Y-X, Zhang Y-D, Shi Y-P. 2023b. Tracking Spatial Distribution Alterations of Multiple Endogenous Molecules during Lentil Germination by MALDI Mass Spectrometry Imaging. Journal of Agricultural and Food Chemistry **71**, 2124-2133.
Zhao D, Yu P, Han B, Qiao F. 2022.) Study on the Distribution of Low Molecular Weight Metabolites in Mango Fruit by Air Flow-Assisted Ionization Mass Spectrometry Imaging. *Molecules* 10.3390/molecules27185873.
